# Ultrastrong Coupling of a Single Molecule to a Plasmonic Nanocavity: A First-Principles Study

**DOI:** 10.1021/acsphotonics.2c00066

**Published:** 2022-03-02

**Authors:** Mikael Kuisma, Benjamin Rousseaux, Krzysztof M. Czajkowski, Tuomas P. Rossi, Timur Shegai, Paul Erhart, Tomasz J. Antosiewicz

**Affiliations:** † Department of Chemistry, University of Jyväskylä, FI-40014 Jyväskylä, Finland; ‡ Laboratoire de Physique de l’École Normale Supérieure, ENS, Université PSL, CNRS, Sorbonne Université, Université de Paris, F-75005 Paris, France; § Faculty of Physics, University of Warsaw, Pasteura 5, PL-02-093 Warsaw, Poland; ∥ Department of Applied Physics, Aalto University, FI-00076 Aalto, Finland; ⊥ Department of Physics, Chalmers University of Technology, SE-412 96 Gothenburg, Sweden

**Keywords:** strong coupling, time-dependent density functional theory, plasmonics, nanophotonics, excitons

## Abstract

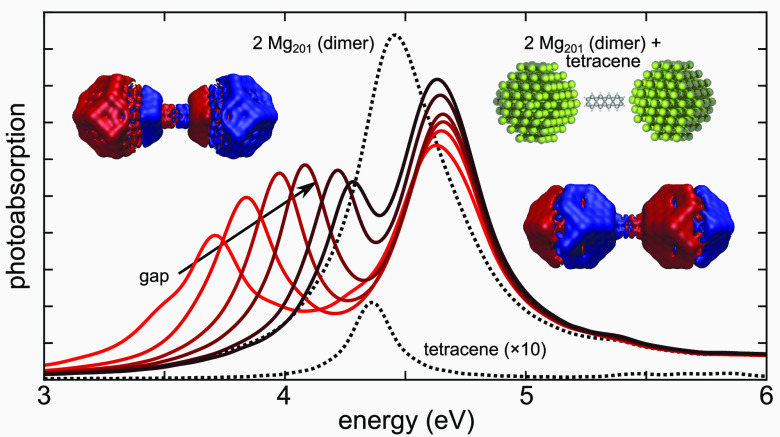

Ultrastrong coupling (USC) is a distinct regime of light-matter interaction in which the coupling strength is comparable to the resonance energy of the cavity or emitter. In the USC regime, common approximations to quantum optical Hamiltonians, such as the rotating wave approximation, break down as the ground state of the coupled system gains photonic character due to admixing of vacuum states with higher excited states, leading to ground-state energy changes. USC is usually achieved by collective coherent coupling of many quantum emitters to a single mode cavity, whereas USC with a single molecule remains challenging. Here, we show by time-dependent density functional theory (TDDFT) calculations that a single organic molecule can reach USC with a plasmonic dimer, consisting of a few hundred atoms. In this context, we discuss the capacity of TDDFT to represent strong coupling and its connection to the quantum optical Hamiltonian. We find that USC leads to appreciable ground-state energy modifications accounting for a non-negligible part of the total interaction energy, comparable to *k*_B_*T* at room temperature.

Strong light-matter coupling is a regime characteristic of interacting systems which can no longer be treated perturbatively. In such a hybridized state the light and matter subsystems are described as dressed polaritonic states with modified behavior that extends beyond optical properties.^1^ These dressed states form when the rate of coupling exceeds the individual damping rates and excitations persist long enough to allow coherent energy exchange between the subsystems. Research in this field^2^ has already led to exciting observations of strong-coupling-induced modifications of exciton transport,^3^ polaron photoconductivity,^4^ photochemical rates,^5^ and ground-state reactivity^6^ as well as single-photon nonlinearities.^7^ For many of these strong-coupling induced phenomena, it is sufficient that the coupling strength is only slightly larger than the damping rates. Commonly, *strong coupling* can therefore be reached already at coupling rates that are much smaller than the resonance energies of the constituent components. By contrast, *ultrastrong coupling* (USC) requires the coupling strength to be comparable to the resonance or transition energies in the case of cavity and matter, respectively.

In the dipole approximation, the magnitude of the coupling strength *g* of cavity-light-matter interaction is determined by the transition dipole moment of a single element of matter μ_1_ = |**μ**_1_|, the number of these elements *N*, and the vacuum field of the cavity *E*_vac_ = |**E**_vac_| according to^1^
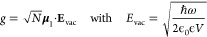
1where *E*_vac_ is evaluated at the quasinormal mode (QNM) frequency ω corresponding to the (complex) mode volume *V*.^8−10^ The strong coupling formalism expressed in the Jaynes–Cummings (JC) Hamiltonian is simple to utilize for interpreting experimental or numerical results when assuming a single cavity mode. However, when two (or more) QNMs are present and may potentially interfere, such as in systems incorporating lossy plasmonic nanocavities, the JC approach is insufficient.^11^ Specifically, it becomes necessary to account for dissipative coupling between the QNMs, resulting in a reduction of the cavity-emitter coupling rate,^12^ and may result in Fano-like interference.^11^ In a single QNM system, however, such as in simple plasmonic systems, in which subwavelength-localized longitudinal fields dominate, eq 1 will remain as the characteristic relation of these systems, such that the effective volume *V* is the volume of a transverse cavity with equal coupling strength,^13,14^ as long as the imaginary part of *V* remains negligible compared to its real part.^10^ When *g* is at least on the order of 10% of the molecular transition energy ω_ex_ (ζ = *g*/ω_ex_ ≥ 0.1), the system is said to be ultrastrongly coupled. An important motivation for reaching the USC regime is a non-negligible ground state (GS) energy modification
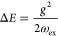
2predicted by cavity quantum electrodynamics (cQED) assuming a single *transverse* optical mode.^15,16^ In the *longitudinal* case, the GS energy modification due to zero-point energy shifts^17^ is attractive and, in the perturbative limit, is known as van der Waals (vdW) attraction. This lowering of the GS energy is in contrast to the repulsive GS energy modification that is obtained in the naïve 2-excitation transverse model, as detailed in the Discussion section in which we summarize this dichotomy. Under favorable conditions, Δ*E* could be on the order of *k*_B_*T* at room temperature, a value which would have significant impact on polaritonic chemistry.^6,18^ By looking at eq 1 and eq 2 one can observe that reaching USC and thus large Δ*E* becomes easier at lower frequencies. Specifically, , while the expected USC-induced GS energy modification is to the first approximation independent of the transition energy, |Δ_e_| ∝ *Nμ*_1_^2^/*V*.

In the pursuit of USC with optical cavities at room temperature, recent work has shown that very large coupling strengths in excess of half of the resonance/transition energy can be obtained even with only 4% of a Fabry–Pérot cavity occupied.^19^ This is possible owing to the very large oscillator strengths of plasmonic nanorods, and the approach can be engineered further to reach even larger coupling strengths.^20^ However, molecules have typically much smaller transition dipole moments than the collective oscillations associated with the conduction electrons in metallic nanoparticles. In the case of molecules, one therefore requires much larger numbers (densities) to reach comparable coupling strengths, typically by filling the entirety of a cavity. Coupling a cavity mode with *N* single-transition molecules leads to *N* + 1 total states, including two (bright) polaritonic states and *N* – 1 purely molecular dark states. The dark states play, however, a crucial role in interactions as they entropically undermine strong coupling. For example, the upper polariton quickly decays into localized dark states.^21,22^ By contrast, in the case of a single molecule (*N* = 1) the number of dark states is zero. This provides a motivation for limiting the number of molecules per cavity.

Here, we explore an alternative approach: Instead of maximizing the coupling strength by using extremely large values of μ_1_ and/or *N*, we reach USC by increasing the vacuum electric field *E*_vac_. A small mode volume *V* on the order of 100 nm^3^ may be obtained in dimers^23^ or particle-on-mirror systems^24^ despite the large size of the employed optical antennas. Even smaller cavities may be reached by utilizing atomic-scale cavities composed of metal clusters with a few hundred atoms, either alone^25^ or arranged in nanoscale dimers,^26^ whose modes can be tuned by the gap size and/or shape.^27,28^ Building on a recent time-dependent density functional theory (TDDFT) study of strong coupling in a benzene-Al_201_ system,^25^ we utilize a cavity made up of two identical plasmonic nanoparticles, resulting in a highly enhanced electric field in a small gap. We then couple the nanoparticle dimer with several different π-conjugated molecules to quantify the dependence of the coupling strength and Δ*E* on the material parameters. We emphasize that the TDDFT approach used here takes into account plasmon decay due to Landau damping and the corresponding dissipation (lower *Q*-values), while electron–electron and electron–phonon scattering processes are taken into account by empirical broadening. This enhances the reliability of the present approach compared to models that rely on two-level systems and/or fully empirical broadening.

Since such an *ab initio* approach allows us to quantitatively investigate longitudinal plasmonic cavities, it is insightful to compare and highlight their differences to transverse cavities. The necessity of the quadratic diamagnetic terms in the transverse Hamiltonian has been discussed in the literature extensively.^29−31^ For example, they protect nongapped systems from infrared divergences^32^ and, in the case of a cavity, offer protection from superradiant phase transitions. Recently Schäfer et al. suggested that the same effects should be present in plasmonic cavities.^31^ Here, we therefore discuss the manifestation of these effects based on quantitative *ab initio* calculations as well as theoretical analysis. We find that they, however, do not emerge upon introduction of coupling per se, but rather result from the *intra*particle effect of plasmonic self-polarization, which also protects from superradiant transitions in interparticle coupling.

## Results

**Figure 1:**
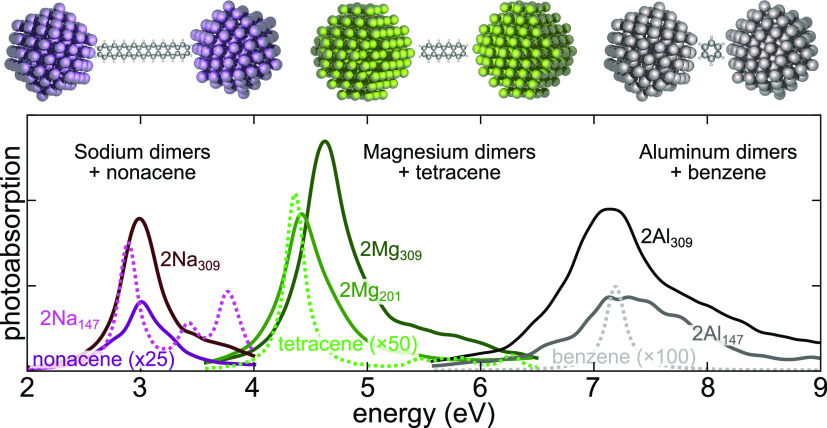
Schematic illustration and photoabsorption spectra of the studied nanoparticle-dimer systems spanning a broad spectral range from visible to UV. From left to right: sodium dimers with 30 Å gap (resonance at 3.0 eV) with nonacene (electronic transition of μ = 15.3 D at 2.9 eV), magnesium dimers with 18 Å gap (4.6 eV) with tetracene (11.1 D, 4.4 eV), and aluminum dimers with 10 Å gap (7.2 eV) with benzene (4.4 D, 7.2 eV).

We model cavities made of sodium (Na), magnesium (Mg), and aluminum (Al) nanoparticle dimers, which exhibit plasmon resonances at approximately 3, 5, and 7 eV, respectively, covering a broad spectral range. These cavities are paired with aromatic hydrocarbon molecules that exhibit highest occupied molecular orbital (HOMO)-lowest unoccupied molecular orbital (LUMO) electronic excitations close to the nanoparticle-dimer plasmon resonances (Figure 1). Their resonance with the respective plasmons ensures adequate spectral overlap even if molecules fill the gap and cause a red shift of the plasmon,^33^ which occurs in parallel to USC. The molecular excitation is tuned to each system by extending the chain from a single ring in benzene to ten rings in decacene. This elongation results in an increase of the transition dipole moment, but simultaneously forces a wider dimer gap to accommodate a longer molecule. Hence, the resulting coupled spectra are determined by a number of factors, some of which are beyond explicit control, as they are dictated by tuning the cavity resonance frequency. All the modeled combinations of dimers, molecules, and spatial arrangements are tabulated in Supporting Information (SI) Table S1, while representative dimer–molecule geometries are displayed in Figure 1 and Figure S1.

Most of the coupled systems considered here feature a single molecule, whose size sets the minimum gap size of the nanoparticle dimer. For example, for a sodium dimer coupled to nonacene the minimum gap is 28 Å. As we will show in the following, these single-molecule–nanoparticle-dimer systems are capable of reaching USC. To gain additional insight into the intricacies of nanoscale polaritons, we also investigate the effects of smaller gap sizes by utilizing closely spaced parallel dimers of shorter molecules. These artificial molecular dimers are designed to have their joint molecular transition at a similar energy as that of a longer molecule. For example, in sodium dimers this condition is fulfilled by two pentacene molecules placed 2 Å apart owing to their mutual interaction. These structures allow us to investigate the limits of USC in nanoscale systems. Furthermore, for selected dimers such as Al_147_ and Mg_309_, we also analyze the impact of nanoparticle orientation on the coupling strength by considering facet-to-facet, edge-to-edge, and corner-to-corner alignments, owing to the fact that such nanoscale details play a crucial role in determining the modal structure of atomic-scale structures.^27,28,34^

### Aluminum

**Figure 2:**
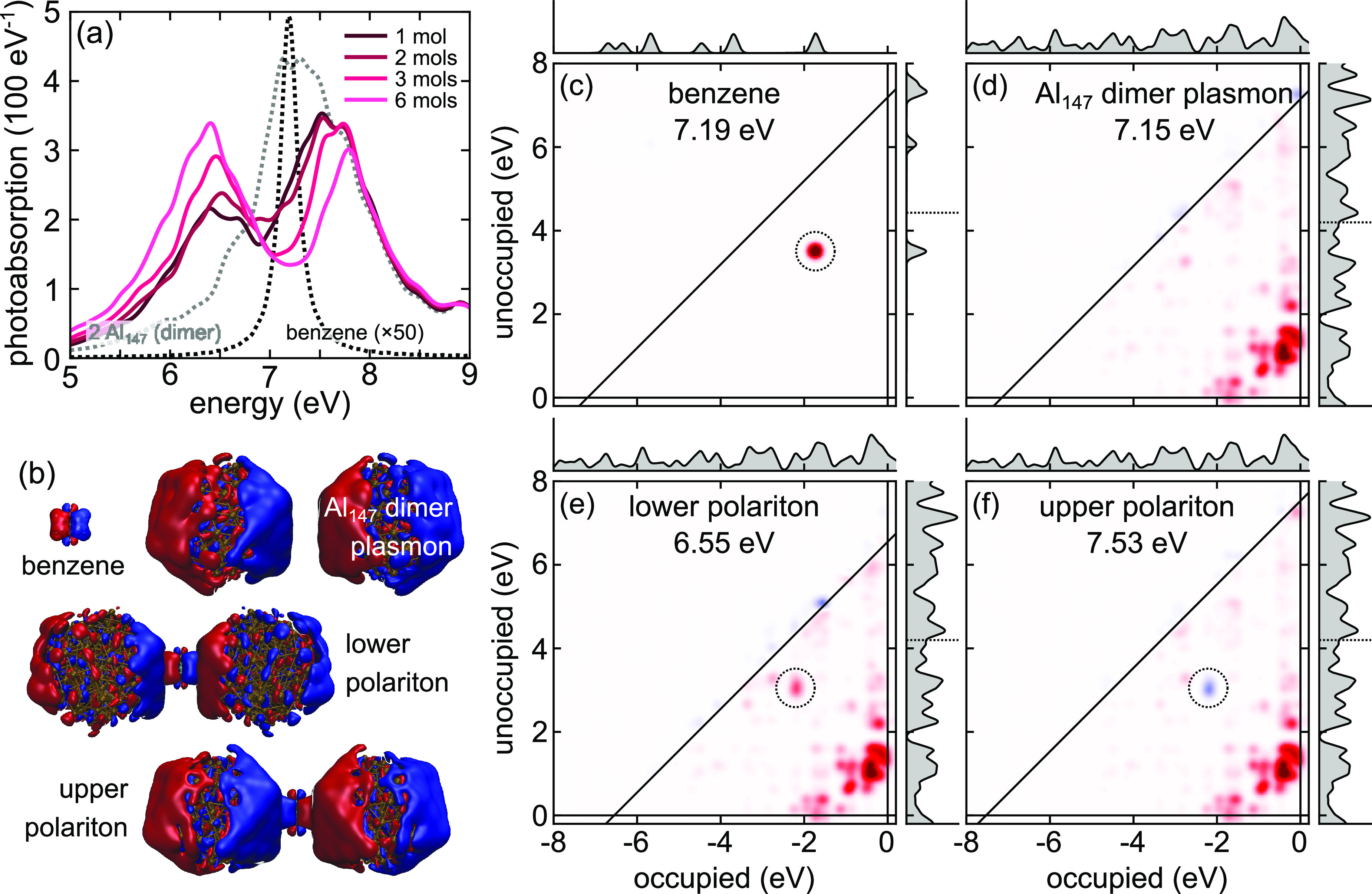
(a) Photoabsorption spectra of Al_147_ dimers with a 10 Å gap coupled strongly to benzene (solid line) and reference spectra (dotted). (b) Induced charge density of benzene, Al_147_ dimer, and the lower/upper polaritons showing the in-phase and out-of-phase combinations. The 3D maps are plotted at the relevant resonant frequencies which are also labeled in the subsequent panels. (c–f) Transition contribution maps (TCMs) of the individual and coupled systems, where red and blue indicate positive and negative contributions, respectively. The TCMs of both polaritons consist of that of benzene and the dimer. At the LP the benzene transition (circled) is in phase with the Al plasmon, while at the UP it is out-of-phase, consistent with the in-phase and out-of-phase nature of LP and UP, respectively.

Figure 2a shows typical TDDFT-calculated photoabsorption spectra, specifically of Al_147_ dimers coupled to a single benzene molecule. The Rabi splitting already for a single benzene is approximately 1 eV and both polaritons become more pronounced with increasing number of molecules. For dimers composed of the larger Al_309_ cluster (Figure S2), despite the same gap size of 10 Å, the splitting of the two peaks is weaker. In fact, for the latter case the spectrum barely exhibits a dip between upper polariton (UP) and lower polariton (LP) with only one molecule involved in the coupling, although for increasing *N* much clearer polaritons develop.

To show that the two observed peaks are indeed the strongly coupled bonding and antibonding of, respectively, the LP and UP, we plot the induced electronic charge densities at the resonances/transitions of benzene, the nanoparticle dimer, and both polaritons (Figure 2b). The dipolar character of the transition in benzene is evident, as is the bright (dipolar) mode of the dimer.^27^ At both polaritons the polarization of the dimer is the same. At the LP and UP benzene is polarized in-phase and out-of-phase, respectively, demonstrating the two orthogonal mixed states of benzene and the dimer.

Further, by analyzing the transition contribution maps (TCMs) (Figure 2c–f), which visualize the Kohn–Sham (KS) electron–hole transition contributions to photoabsorption.^35^ The benzene transition at 7.19 eV is doubly degenerate with an energy difference between the occupied and unoccupied states of approximately 6.5 eV, while the Al_147_-dimer plasmon consists of a large number of coherent transitions from just below to just above the Fermi energy with a predominant energy difference of approximately 2 eV. The TCMs of the two polaritons, which are formed by mixing of the molecular exciton and plasmon, show the same characteristic distribution of the KS transitions.^25^ However, at the LP the benzene transitions are in-phase with those of the plasmon, while at the UP they are out-of-phase, hence screening the plasmon. These observations are qualitatively identical for other studied systems.

### Sodium

**Figure 3:**
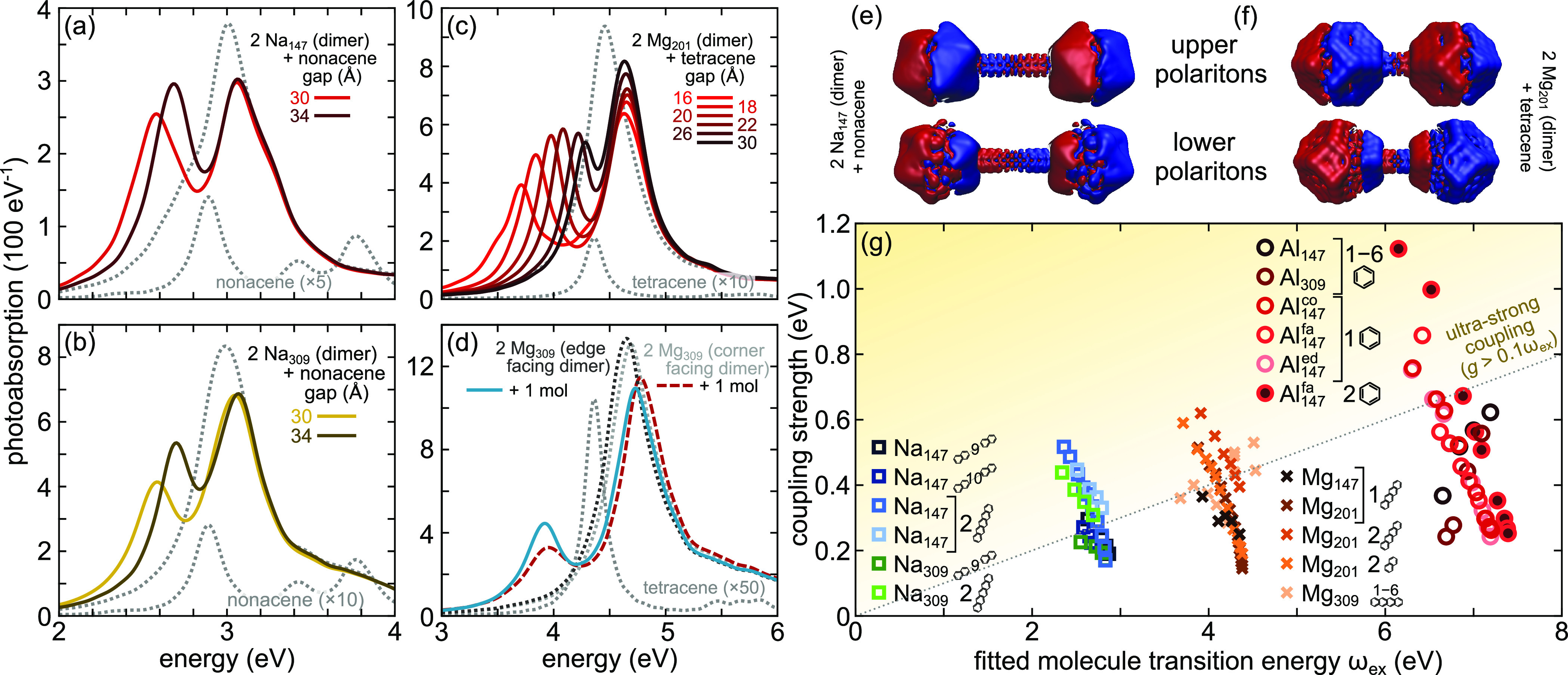
Photoabsorption spectra of strongly coupled molecule–nanoparticle-dimer systems (solid lines, gray dotted lines mark reference spectra): (a,b) Na nanodimers coupled to nonacene (reference Na dimer gap 30 Å), (c,d) Mg nanodimers to tetracene (reference Mg dimer gaps 18 Å). (e,f) Induced charge densities at the lower and upper polaritons for Na and Mg dimers coupled to molecules showing the bonding and antibonding character of a strongly coupled system. (g) Fitted coupling strength *g*_fit_ for all systems versus the fitted molecular transition energy ω_ex_. The results are arranged in three groups: Na dimers (squares), Mg dimers (crosses), and Al dimers (circles). The insets in the legend mark the different cases; for a single shape/color the varying parameter is either the gap size (continuous variation of points) or the number of molecules (scattered points). The dotted line marks 0.1ω_ex_, the commonly adopted lower limit of USC. The coupling strengths vary significantly with gap size. For all nanoparticle dimers the USC regime can be reached through a suitable combination with molecules.

Figure 3a,b presents additional photoabsorption spectra of selected coupled systems (also see Figure S2), clearly showing LP and UP. For the lowest energy case, sodium Na_147_ dimer in Figure 3a, the Rabi splitting is on the order of 0.5 eV for the smallest dimer gap with LP and UP being of equal amplitude, indicating only minor detuning between the molecular and Na_147_ resonances. In Figure 3e we show the corresponding induced charge densities. For the coupled Na_309_ dimer (Figure 3b), the UP is more pronounced than the LP due to larger detuning, although the plasmon peak positions are similar in Na_147_ and Na_309_. The similar Rabi splitting in these systems indicates that the coupling strength is sensitive to the gap size but not to the size of the nanoparticles.

### Magnesium

**Figure 4:**
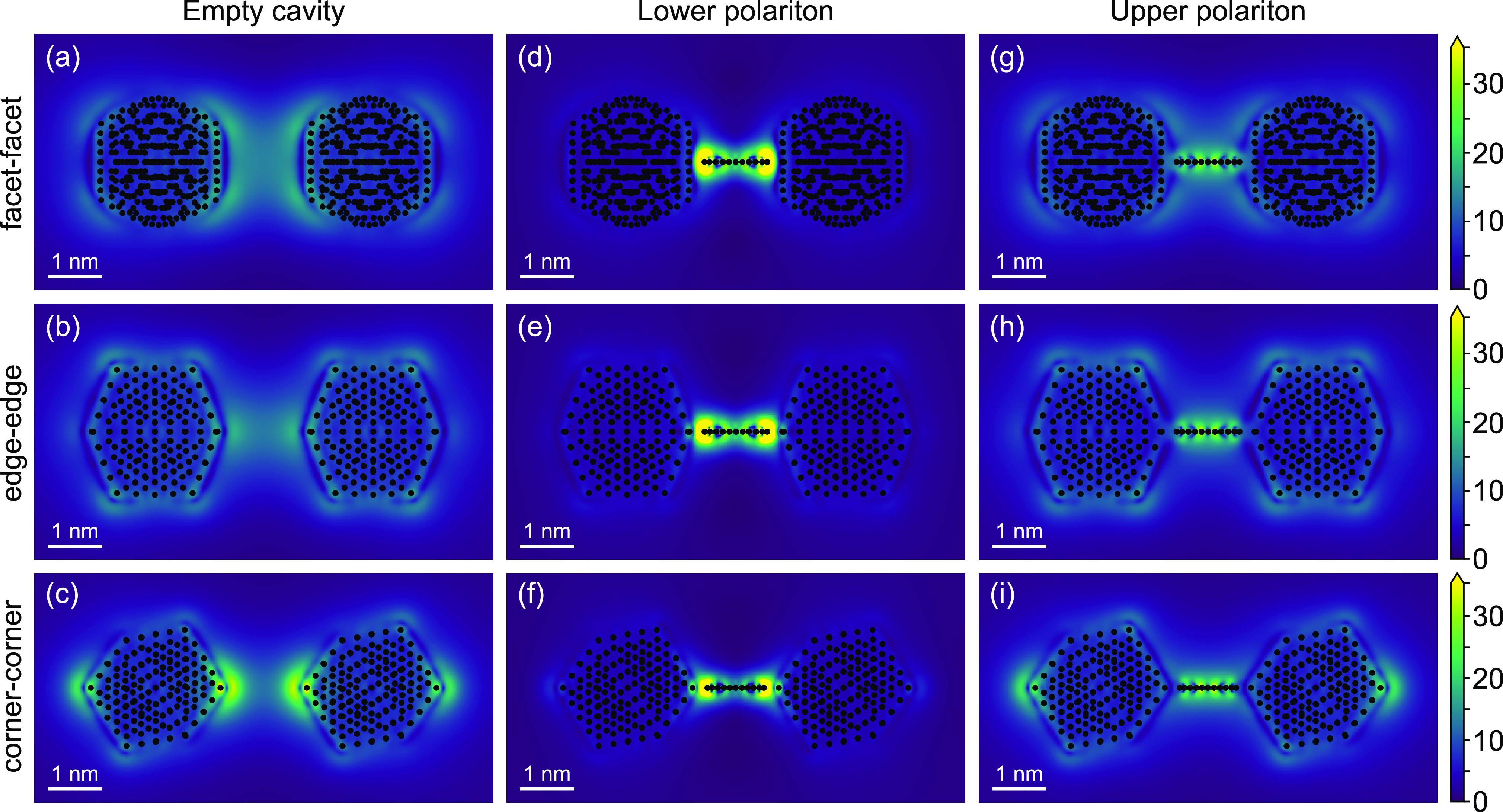
(a–c) The electric field enhancement of empty Mg_309_ dimers with 18 Å gap differs qualitatively with the relative orientation of the nanoparticles, including significant quantitative differences in their maximum amplitudes. In contrast, coupling to tetracene, leads to induced fields at (d–f) lower polaritons and (g–i) upper polaritons that are qualitatively similar, which illustrates a significant modification of the cavity by the presence of the molecule that occurs in addition to the USC of the molecular electronic transition to the nanoscale-dimer plasmons.

In Figure 3c, Mg_201_ dimers are coupled to tetracene with smaller gaps than in the Na dimers, while a plot of an exemplary induced charge density at the LP/UP is shown in Figure 3f. The coupling strength for the smallest gap is 0.6 eV, a significant fraction of the 4.4 eV transition energy. For the larger Mg_309_ dimer with tetracene (Figure S2) the coupling strengths are up to 15% smaller than for Mg_201_ for the same gap size, which is in line with the larger mode volume of Mg_309_. Additionally, the coupling strength should be affected by the relative orientation of the dimer components. For example, a dimer in corner-on-corner orientation localizes the electric field more strongly than the facet-on-facet configuration (Figure 4a,c),^27^ which suggests that a single-atom protrusion may be considered a picocavity.^34,36^ Also, at the level of the empty dimers one observes slightly different plasmon energies for larger gaps as well as the appearance of a charge transfer plasmon in addition to the bright dipolar mode.^34^ Combined, these differences between the differently oriented dimers result in different localization of the modes with variations by up to a factor of 4^27^ with the induced electric fields shown in Figure 4a–c. Such differences should theoretically result in coupling strengths varying by as much as 50%. This is, however, not observed. Indeed, the Rabi splittings in all three dimer orientations are nearly identical when coupled to tetracene (Figure 3d). The only differences are small variations of the relative amplitudes of the LP and UP, which are caused by the slight differences between the cavity resonance energies and different detuning with respect to the electronic transition of the molecule. The origin of the equal coupling strength in all three cases is made clear via the induced electric fields of LPs and UPs, especially in the gaps. Despite the different orientation of the dimer elements, both polaritons are quantitatively very similar (Figure 4d–i), demonstrating that in such coupled systems the molecule changes the character of the cavity, overriding the unique individual modal distributions when forming hybridized modes. Thus, the picoscale electric field localization offered by single atoms^34,36^ may disappear with increasing coupling, resulting in a mode volume that is determined by the molecule(s) rather than the atomic features of the metal antenna(s).^23,37^

### Single-Molecule USC

The photoabsorption spectra are fitted with the velocity-coupled harmonic oscillator model, Methods section and eq 7, to obtain the coupling strengths *g*_fit_ (fitting parameter), which are plotted in Figure 3g as a function of the fitted molecular excitation energy ω_ex_ (see Figure S3 for per-molecule coupling strength). Grouped by cavity material from lowest to highest resonance energy, the results show that very strong interaction is possible even with a single molecule occupying the gap. When a single molecule is replaced by a pair of molecules (an artificial molecular dimer; Figure S1d,f) to enable the use of smaller gaps, *g* becomes even larger. Already the coupling strengths of the single-molecule cases exceed 0.1ω_ex_ (dotted line), reaching similar values (ζ = *g*/ω_ex_) for all three systems in the range 0.11 to 0.13. For two molecules in the gap ζ is even larger, reaching ζ = 0.22 for Na, ζ = 0.18 for Al, and ζ = 0.16 for Mg (see Supplementary Table S2).

## Discussion

### Effective Vacuum Fields

In the strong coupling regime, and especially in the USC limit, discussing the system in terms of undressed molecules and bare dimers is not appropriate, since the underlying components are thoroughly mixed into the new polaritonic states. This is demonstrated by the qualitative similarity of the field distribution at LP and UP (Figure 4) in different configurations, as local atomic-scale variations in the individual components become much less pronounced in the coupled systems. To gain intuition, it is instructive to refer to the basic characteristics of a strongly coupled system as expressed via eq 1, by calculating the effective vacuum fields and comparing them to the theoretical values (see Methods) of the corresponding empty dimers. The latter requires an evaluation of the volume of the corresponding QNMs.^8^

For our discussion we define the *effective* vacuum field *E*_vac_^eff^, based on the coupling strengths *g*_fit_ obtained from fitting the spectra with the coupled harmonic oscillator model in eq 7, as the magnitude of the electric field necessary to obtain *g*_fit_ when acting onto the transition dipole moment of *N* molecules coupled to the dimer. It is calculated as
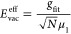
3based on eq 1. Thus, *E*_vac_^eff^ accounts not only for the inhomogeneous field of the plasmonic cavity, but also for other coupling-induced modifications of the interacting dimer-molecule system that are not captured by simple models. The additional effects include contributions from higher energy transitions of the molecule that reshape the cavity mode, the interaction between KS states of the interacting entities or shift of the cavity resonance.

As TDDFT deals directly with electronic properties of matter rather than derived optical quantities such as permittivities, direct use of the existing computational QNM^38^ formalism is not feasible. Hence, to compute the QNMs as outlined in ref (39) (see Methods for additional details), we approximate the atomic clusters by Drude nanospheres whose permittivity is tailored to match the peak position, width, and amplitude obtained with TDDFT and the radius is determined by the physical size of the cluster (see Table 1 for parameter values). This allows us to subsume all nonlocal and quantum size effects^40^ into an effective permittivity, which is a good approximation for the auxiliary role of employing the QNMs to verify the effective vacuum fields obtained from TDDFT calculations and *g*_fit_. The mode volumes, being a position-dependent quantity, are calculated assuming the probing dipole is placed parallel to the dimer axis in the geometrical center of an empty dimer gap. To account for a finite size of the molecules, the dipole is displaced from the center to 1/fourth of the gap size or at the position of the end of a molecule. Next, we use the QNM volumes (Figure S7) to calculate the corresponding QNM vacuum field *E*_vac_^QNM^ that acts on a point-like molecule. The imaginary parts Im{*V*} of the QNM volumes are also shown to be at least ten times smaller than the real parts Re{*V*}, Figure S8).

**Table 1:** Parameters Used in the Classical Electromagnetic Calculation of the Quasinormal Modes of Nanodimers: Nanosphere Radius, Drude Permittivity: ϵ(ω) = 1 – ω_*p*_^2^/(ω^2^ + *iγω*)

structure	radius (Å)	ℏω_*p*_ (eV)	ℏγ (eV)
Al_147_	7.4	13.62	0.75
Mg_201_	9.8	5.30	0.47
Na_147_	10.1	8.03	0.47

**Figure 5:**
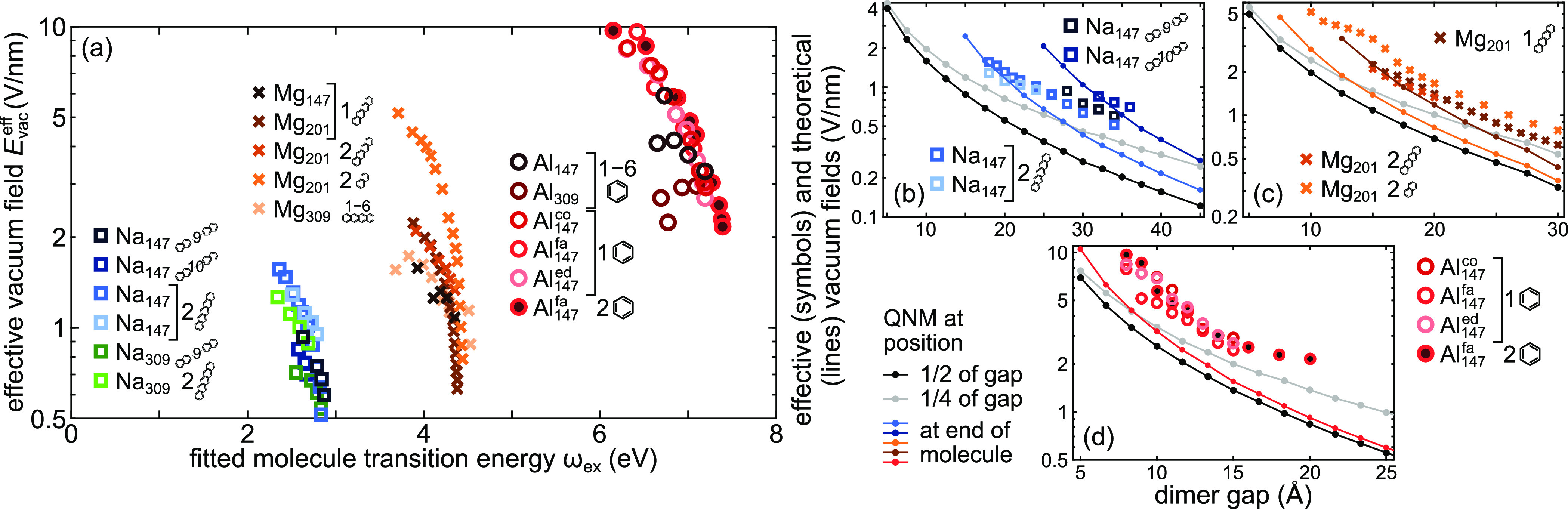
(a) Effective vacuum field *E*_vac_^eff^ of the dimer cavities interacting with the molecules for Na (squares), Mg (crosses), and Al (circles) for various molecules and gap sizes. A larger *E*_vac_^eff^ for the same colored symbol typically corresponds to a smaller gap. For Na the effective vacuum field is small and does not vary by a lot, being a consequence of large gap sizes and small overlap between mode and molecule. This applies also for the systems involving Mg, except for a Mg dimer coupled to a naphthalene dimer, for which *E*_vac_^eff^ changes significantly. For Al the vacuum field spans a broad range of values. (b,c,d) Comparison of effective vacuum fields (symbols) of dimer-molecule systems with the QNM vacuum field (lines) of empty dimers versus gap size with the probing dipole placed in the center of the gap (1/2, black) or displaced toward one of the dimer components to 1/4 (light gray) of the gap. The colored lines mark vacuum fields of empty dimers for dipoles placed at an end of the corresponding molecule (whose color in the plot matches the color of the line).

The computed effective vacuum field values are plotted in Figure 5a as a function of the fitted electronic transition energy of the molecule. *E*_vac_^eff^ approaches 10 V·nm^–1^ for Al_147_, 5 V·nm^–1^ for Mg_201_, and 2 V·nm^–1^ for Na_147_. The ratio of the maximum and minimum effective vacuum fields for the three metals is approximately 20 and is markedly larger than the corresponding ratio for the coupling strengths, which approaches 6. This can be rationalized by the competition between a decreasing mode confinement when going from Al to Mg to Na in order to accommodate increasingly longer molecules with an increasing transition dipole moment. This decrease in mode confinement with increasing gap is clearly visible Figure 5b–d, where we compare the effective and theoretical vacuum fields.

In general, the vacuum fields corresponding to the bright dipolar bonding QNMs, *E*_vac_^QNM^, increase for decreasing gaps, as expected with the probing dipole moving closer to the nanoparticles. They also increase for a fixed gap, when the dipole is brought closer to one of the dimer components. For all three Na, Mg, and Al dimers the vacuum fields are similar for any given gap size and span a range from ca. 0.2 to 7 eV, although the latter’s *E*_vac_ is the largest of these three. This is, however, mainly caused by an increase of the frequency of the QNM, since for a given gap size the QNM volumes of all dimers are similar (Figure S7). Also, due to the very small size of the particles (ca. 1 nm), any higher order modes are much weaker and their mode volumes are a few orders of magnitude larger yielding negligible interaction with the molecules/dipoles.

The effective vacuum fields, which are derived from the fitted coupling strength, are larger than the ones based on the QNMs regardless of the probing position, be it in the middle of the gap or at the position of the end of the molecule. This demonstrates the complex interactions that clearly go beyond a simple two-by-two coupling model, highlighting the fact that the molecule itself reshapes the cavity mode. This further is demonstrated in Figure S4, which shows the TDDFT-calculated field profiles for Mg_201_ dimers with gap sizes of 15 and 30 Å, respectively (Supplementary Note S1). The average energy density of the induced electric field in a volume corresponding to a centrally placed molecule for the 30 Å gap is on the order of 10% of the maximum value (observed near one of the Mg atomic clusters). However, the ratio of *E*_vac_^eff^/*E*_vac_^QNM^ is approximately 2 for both one tetracene and two naphthalene molecules. For the smaller gap of 15 Å, the average normalized field intensity is ≈0.4, while in the coupled system the ratio *E*_vac_^eff^/*E*_vac_^QNM^ is 2 for tetracene and 3 for two naphthalenes. Clearly, the molecules couple to the cavity mode with greater efficiency than predicted by the vacuum field of the bare cavity and position of the molecule.

The interaction in such nanoscale polaritonic systems extends beyond coupling the cavity mode to the electronic transition, as it also involves a non-negligible modification of the cavity and facilitates reaching USC (Figure 4). Indeed, based on our findings we ascertain that the cavity is modified by electronic states, which are not involved in the molecular HOMO–LUMO transition that couples directly to the plasmon. Molecules not only couple as dipolar resonators but form a kind of dielectric bridge focusing the field of the cavity mode and increasing the effective coupling in the system. This modification of the cavity beyond a two-level model by the *body* of a molecule is reminiscent of the effect of background permittivity of a quantum dot interacting with a plasmonic bow-tie antenna.^23^

### USC Contribution to Ground State Energy Shifts

We now estimate the ground state energy shifts in the USC regime. As in recent quantum electrodynamics DFT (QEDFT) calculations,^41^ which require the quadratic diamagnetic or self-polarization terms to be accounted for consistency,^31^ in our case also, analogous terms appear and ensure consistent results as discussed in the previous section. Hence, the computed spectra and underlying coupling strengths are sound, as is the case also in classical calculations,^19^ and the results can be interpreted in the framework of quantum mechanical Hamiltonians, namely the Hopfield model.^15^ One of the results of the Hopfield Hamiltonian is a predicted modification of a system’s GS due to admixing states with higher numbers of excitations, which in the USC regime may constitute a significant fraction of *k*_B_*T* at room temperature.^19,20^ However, due to the small cavity sizes in our study, we use the longitudinal Hamiltonian as derived in Supplementary Note S2 as a simple model to interpret the results to an adequate approximation. This is justified since the imaginary parts of the mode volumes (responsible for non-Hermiticity and dissipative couplings) were shown to be at least ten times smaller than the respective real parts (Figure S7). The equivalence of this model to TDDFT is discussed in the following section with technical details presented in Supplementary Note S3. At zero cavity-exciton detuning the resulting shift of the ground state energy is  (Supplementary Note S2). We emphasize that our calculated longitudinal zero-point energy shifts are concrete random phase approximation (RPA)-correlation energies describing vdW forces in a low coupling limit as discussed in Supplementary Note S2.

**Figure 6:**
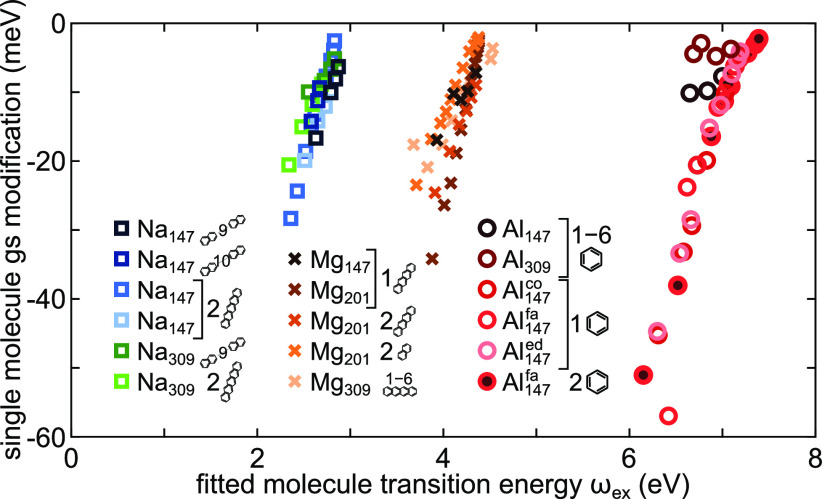
Predicted ground state energy modification based on cQED estimate in the single molecule USC regime is on the order of *k*_B_*T* at room temperature for all considered systems. Al dimers yield the largest GS of up to 60 meV, but this is observed only for gaps ≲10 Å, in which the large coupling strength is obtained by a combination of small gap size and molecule-induced modification of the mode volume. In Mg and Na dimers the predicted GS modification is smaller than for Al dimers.

Using the fitted coupling strengths (Figure 3e), the longitudinal zero-detuning cQED result is used to calculate the expected GS modifications (Figure 6). The calculated  are on the order of a few tens of meV, values which constitute a significant fraction of *k*_B_*T* at room temperature. The maximum  of up to −60 meV is predicted for Al owing to the very large coupling strengths, especially for gaps on the order of ≲10 Å. For Mg and Na the expected GS modifications are smaller by a factor of 2 due to a smaller coupling strength which is not fully offset by their respective lower transition energies. While these numbers are small in comparison to the unperturbed ground state energies, they are obtained for a single molecule coupled to an optical cavity. Although our cavities are model systems, they are still based on ab initio TDDFT and representative for what occurs in particle-on-mirror geometries^24^ or picocavities^36^ and these results can provide estimates for the order of magnitude of USC modifications to the ground state energy landscape. One of the relevant impacts of these changes is modification of ground state chemical reactivity via USC^42^ and correspondence to vdW forces.^43^ We thus look into the energy scales of these interactions, keeping in mind that USC modifications calculated here refer to a single optical mode correction, while vdW accounts for all possible modes and polarizations.

**Figure 7:**
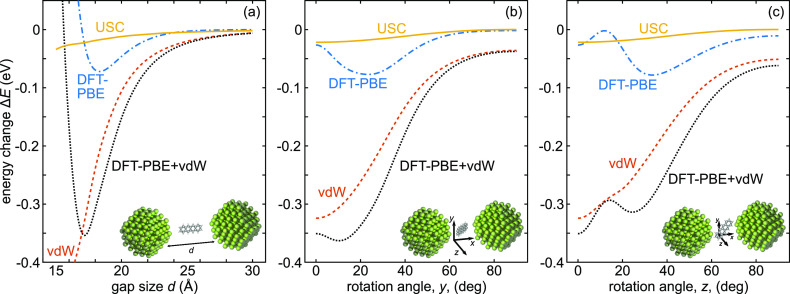
Comparison of predicted ground state energy modification in the single molecule USC regime based on the longitudinal quantum optical Hamiltonian (solid orange line) with other contributions to the energy landscape, namely DFT-PBE (dashed-dotted blue line) and vdW (dashed dark orange line), for a Mg_201_ dimer coupled to tetracene as a function of (a) dimer gap size, (b) *y*-axis rotation angle of tetracene, and (c) *z*-axis rotation angle of tetracene. The gap size in (b,c) is 17.2 Å, the minimum of the energy landscape in (a). In general, the USC energy modification plays a small part in modifying the energy landscape, but due to the longitudinal coupling its contribution results in a more stable system.

We compare USC modifications and vdW for Mg_201_ dimers with a single tetracene molecule. In addition to the USC GS modification, we calculate the total energy contributions as a function of three parameters: gap size and tetracene rotation angle (see Figure S5 for *g*). The largest contribution is calculated with conventional (static) density functional theory (DFT) using the *semilocal* PBE^44^ exchange-correlation functional. As PBE does not include vdW interactions, which are associated with *nonlocal* correlation,^45^ we evaluate their contributions to the energy by calculating dispersion corrections using the DFT–D3 method.^46^ We use DFT–D3 as opposed to a nonlocal functional such as vdW-DF^45^ as it provides a simple means for quantifying the magnitude of the vdW contribution, enabling direct comparison with the magnitude of the USC contribution. The energy contributions from DFT-PBE and vdW are calculated as Δ*E* = *E*_2Mg_201_+tetr_ – *E*_2Mg_201__ – *E*_tetr_ (Figure 7).

First, we evaluate the energy as a function of the gap size *d* and separate the individual contributions from DFT-PBE and vdW, as well as the USC estimation (Figure 7a). The sum of DFT-PBE (Δ*E*_DFT_) and vdW (Δ*E*_vdW_) shows that the Mg_201_-tetracene-Mg_201_ system is bound at *d* = 17.2 Å with a potential well of about 350 meV. The USC correction is small amounting to approximately 30 meV, which is only 1 order of magnitude smaller than the other two terms. Thus, with respect to distance, USC has a small impact on the total energy at equilibrium and if separated out, it would be responsible for a small shift of the potential well on the order of single picometers. However, the USC correction decays less rapidly than Δ*E*_DFT_ or Δ*E*_vdW_ with the gap size, reaching 20% of their joint value (Figure S6k). Thus, a single strongly coupled mode can account for a significant portion of the full vdW interaction.

Next, we consider the rotations of the tetracene molecule in the gap. The center of the molecule is fixed to the middle of the gap with *d* = 17.2 Å (Figure 7b,c). For rotations about both *y* and *z* (see inset of Figure 7b,c), Δ*E*_vdW_ is smallest when the molecule is aligned along the dimer axis. Δ*E*_DFT_, on the other hand, has different minima for the different rotations. For the *z*-axis rotation, the energy also shows a local maximum caused by corner hydrogen atoms moving closer to Mg during rotation. The Δ*E*_USC_, however, is an order of magnitude smaller at −30 meV at 0° rotation angle and can only slightly change the angular positions of the minima.

We also calculated the rotation-dependence for other gap sizes (Figure S6). Overall, the contribution of the single mode USC to the GS is small, typically between 5–20% of the total energy. The largest relative values occur for larger gaps when Δ*E*_DFT_ and Δ*E*_vdW_ are small, or for small gaps when Δ*E*_DFT_ and Δ*E*_vdW_ cancel each other out. However, overall the largest potential impact of USC on the complex energy landscape occurs for large *g* which take place for small gaps.

In the studied cases the USC modifications to the ground state can reach significant values. Hence, one may ask if this fraction could be larger in other nanoscale systems. Overall, it is a question of balancing repulsive and attractive forces vs distance, rotation angle, and potentially molecule number and confronting these with the achievable ultrastrong coupling. A simple answer is not straightforward as the number of parameters to investigate/optimize is very large and, to a large extent, all Δ*E* contributions are susceptible to any of the above parameter changes. A key point to be addressed further, is the possibility of decoupling the induced electric field responsible for USC from the structural influences on PBE and the rest of vdW energy changes. Such decoupling would allow one to modify USC semi-independently of Δ*E*_PBE_ and Δ*E*_vdW_, although presently it is unclear to what extent it would be achievable. However, based on these initial studies, enhancing USC to significantly modify the energy landscape of nanoscale systems in the single-molecule regime at the scale of 100 meV appears challenging.

### Relation between TDDFT and Longitudinal Hamiltonian

In the previous section, we employed the coupling coefficients *g* obtained by TDDFT to estimate the shift in the ground state energy using the correction that is obtained from a simple Hopfield Hamiltonian. This assumes that the *g* values obtained by TDDFT carry the same physical meaning as the coupling coefficient that enters the Hopfield Hamiltonian. It is thus warranted to discuss the validity of this approximation.

TDDFT is an exact theory that captures the dynamics of any electronic Hamiltonian, where the electrons are coupled only longitudinally via the Coulomb potential.^47^ To the extent that we can neglect transverse photonic modes in the nanoscale dimers considered here, our calculations therefore provide an exact model up to the exchange-correlation approximations used. This also implies that the cQED extension of DFT/TDDFT to transverse photons is not required.^31,41^

It is not straightforward to compare model optical Hamiltonians rooted in second quantization with TDDFT, which is a classical field theory derivable from a classical Lagrangian operating on auxiliary densities. There is, however, plenty of common ground, as the TDDFT equations of motion may be written using classical mechanics with Poisson brackets.^48^ Also, it is customary to bosonize the Fermionic polarization, which allows one to approximate a polaritonic system as a quadratic bosonic Hamiltonian. Bohm and Pines^49^ applied this approach to the collective degree of freedoms (DOFs) in metals, establishing the foundation of the modern RPA, while Hopfield^50^ used it for the treatment of localized dielectric response. These two examples represent the two extremes: free electrons associated with a plasmonic current and confined localized polarization.

Thus, the common denominator is the symplectic structure and similar linear equations of motion derivable either with commutators or Poisson brackets. There is thus a relation via the RPA, which corresponds to the TDDFT-exchange correlation (XC) kernel being zero *f*_xc_ = 0 and allows one to write the RPA-TDDFT equations of motion in second quantization.

To recognize the relation between model optical Hamiltonians and TDDFT it is useful to recast the latter in a similar 2 × 2 form as the Hopfield Hamiltonian. A suitable starting point is the Casida (frequency space) formulation of TDDFT.^51^ In this approach the response of the system is obtained by solving the following equation (see Supplementary Note S3)
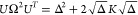
4where *U* is an orthonormal matrix diagonalizing the Casida matrix, and Ω is a diagonal matrix containing Casida eigenvalues, Δ are the electron–hole excitation energies, and *K* represents the coupling between the excitations. To obtain an estimate of the magnitude of *K*, which corresponds to the self-polarization energy, neglecting dissipation (including hot carrier generation^52^), we can transform the system such that each dipolar plasmonic mode is represented by a 1 × 1-block in the Casida form . Since Δ_P_ is a weighted sum of very low energy electron–hole transitions contributing to the (collective) plasmon, Δ_P_^2^ ≈ 0, and one is left with
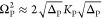
5For nanoparticles in the size between approximately one hundred and 1000 atoms, the average of the Kohn–Sham excitations Δ_P_ is on the order of 1 eV and decreases with increasing size.^25,35,52^ The plasmon energy on the other hand is Ω_P_ = 3.7 eV for Ag^35,52^ and Ω_P_ = 7.8 eV for Al.^25^ This gives self-polarization energies *K*_P_ of 7 and 30 eV for Ag and Al, respectively. Although the magnitude of Δ_P_ decreases for large nanoparticles, the plasmon energy remains large and finite. We can thus represent the coherent low energy KS excitations as a single plasmonic mode, which we can identify as being analogous to the cavity element of the Hopfield Hamiltonian. These modes can then be connected to lowest order via dipolar coupling, yielding an equivalent form to the Hopfield Hamiltonian. This provides the basis for the dipolar coupling of subsystems, specifically nanoparticles and molecules, where the individual components are already diagonalized TDDFT systems.^53^

We note that in the bulk limit (*q* → 0) Δ_P_ goes to zero linearly in *q* while *K*_P_ diverges as 1/*q*^2^. Ω_P_ does not, however, diverge due to the presence of noninteger occupation numbers (which were omitted in eq 5 above) and approaches the bulk plasmon. This is equivalent to the protection against the infrared divergence familiar from Hopfield Hamiltonians.^31^

In Supplementary Note S3, we start from the RPA second quantization Hamiltonian (excluding exchange) of two subsystems (for example two plasmonic nanoparticles or an ultrastrongly coupled molecule and a nanocavity), and diagonalize the subsystems. At this stage, the intrasystem correlation energy of the subsystems may be obtained. This demonstrates a key difference between the self-polarization in the transverse system, where it is introduced due to coupling, and the plasmonic self-polarization here, which occurs regardless of intersystem coupling and is not caused by it. With this setup, we heuristically demonstrate a mechanism for a similar “no-go” theorem for the superradiant phase transition in purely longitudinal plasmonic systems, as Bernardis et al. presented for a two level system for a particular gauge.^54^ Here, we discuss this in terms of coupling of a single plasmon to a molecule. Most of the oscillator strength of the plasmon, which is due to coherent collective excitations of intraband electrons (∼1 eV KS energy) in the subsystems, ends up in the plasmon of energy Ω_P_.

Strengthening of the interaction between a cavity and a molecule requires more oscillator strength [see eq 1]. As a result of the Thomas–Reiche–Kuhn-sum rule,^55,56^ more oscillator strength requires a larger number of occupied levels. This increases the electron density in a confined volume and pushes the plasmon energy upward due to self-polarization, thus protecting the systems from a superradiant phase transition upon introduction of intersystem coupling. This is demonstrated in the SI by making a canonical transformation to the subsystems, and observing their dipolar coupling at the new renormalized energies. This is analogous to the transverse cavity case, where the *g*_*c*_^2^/ω_*m*_(*â* + *â*^†^)^2^ term protects from a superradiant phase transition,^19^ and equivalently, this positive shift (renormalization) may be incorporated in the cavity eigenvalues
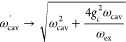
6by means of a Bogoliubov rotation.^15^ Here, the canonical transformation to the TDDFT subsystems is in analogy to the Bogoliubov transformation (which is also a symplectic transformation). After we have diagonalized the subsystems, i.e., obtained a strong dipolar plasmon, we diagonalize the full system and obtain the zero-point energy shifts corresponding to the vdW interaction. Thus, we note that the zero-point energy shifts due to plasmonic self-polarization should not be mistaken for intersystem effects. This is in contrast to transverse coupling, where the *A*^2^ term is introduced upon the coupling itself (care must be taken here as well in a single cavity mode approximation, since in a cavity, compared to free vacuum, there is not only an extra cavity mode, but absence of free vacuum modes below the plasmonic cutoff of the cavity).

## Conclusions and Outlook

In this work, we have explored the possibility of reaching the ultrastrong coupling regime with single molecules by coupling them to idealistic optical plasmonic nanocavities. Our calculations indicate that single-molecule-based USC is viable across a broad spectral range, but requires balancing the size of the optical mode and its vacuum electric field with the size and transition dipole moment of the molecule. Herein, we set the plasmonic cavity sizes and their resulting QNM volumes to the range of 1 to 10 nm^3^. This ensured good matching with the size of the molecule, allowing for very efficient use of the enhanced vacuum field. Specifically, for Mg_201_ and Al_147_ dimers with the smallest possible gaps, the mode volume of coupled systems approaches 1 nm^3^, which is close to the size of the molecules. In fact, squeezing the mode volumes even further could become counterproductive for reaching single-molecule USC, since single-atom picocavities^34,36^ could couple to individual atoms in nearby molecules, rather than the entire molecule, and induce, e.g., local modifications of the subgroups of a molecule.

Simultaneously, sub-10 nm^3^ mode volumes are associated with very large vacuum fields, here calculated to be in excess of 10 V·nm^–1^. It is only in such large fields that the USC regime can be reached with a single molecule. Furthermore, the ultrastrongly coupled dressed light-matter systems studied here clearly manifest effects that extend beyond two-level models. This pertains specifically to the modification of the cavity by higher-energy excitations of the molecule, which contribute to the dielectric response of the molecule. They focus the mode in the vicinity of the molecule and increase the coupling strength, inducing shifts of the resonant energies of the components of the system. For comparison of magnitudes, real electric fields of comparable amplitude are obtained with attosecond lasers with peak intensities on the order of or exceeding 1TW·cm^–2^. Naturally, the vacuum fluctuations in ultrasmall cavities should not be mistaken for real fields, but it might be interesting to study their implications in the future with TDDFT beyond the linear response regime, including cQED in the computational framework.^41^

In summary, the good matching of the spatial dimensions of the cavity and coupled molecules as well as the cooperation of the vacuum field and transition dipole moment of the molecules can enable single-molecule USC across the entire studied spectrum. For Al nanoparticles the dominant contribution responsible for their USC to benzene comes from the very strong vacuum field, which can exceed 10 V·nm^–1^. Mg and Na nanoparticles exhibit, respectively, gradually weaker vacuum fields and rely more on the increasing transition dipole moment of the molecule to reach USC. However, even for Na dimers the vacuum fields can easily exceed 2 V·nm^–1^. These enable single-molecule coupling strengths on the order of 13% of the molecular excitation energy. In turn, the predicted USC ground state energy modification estimated by using cQED reaches 30–60 meV for a single molecule, which is comparable to *k*_B_*T* at room temperature. Such ground state energy modifications could have significant implications for strong-coupling assisted chemistry and other material properties.

## Methods

### DFT and TDDFT Calculations

The DFT and TDDFT calculations were carried out using the PBE^44^ exchange-correlation functional in the adiabatic limit. The photoabsorption spectra are calculated using the δ-kick technique^57^ in the linear-response regime and employing the dipole approximation for light-matter interaction. The spectrum is presented as the dipole strength function that is equivalent to photoabsorption cross section apart from a constant multiplier. The default projector augmented-wave (PAW)^58^ data sets and double-ζ polarized (dzp) basis sets provided in GPAW were used for Al, C, and H. The dzp basis set of Al includes diffuse 3p functions, which are important for describing plasmon resonances.^59^ For Na and Mg the corresponding p-valence basis sets were used with only one and two, respectively, electrons in the 3s orbital considered explicitly, while the lower electrons were treated as a frozen core within PAW. This simplification had only a minute impact on the photoabsorption spectrum as verified against the larger PAW setups. In general, while the used basis sets might not be adequate for yielding numerical values at the complete-basis-set limit, they are expected to be sufficient for the purposes of the present work. For real-time TDDFT a grid spacing parameter of 0.3 Å was chosen to represent densities and potentials, and the molecules/particles were surrounded by a vacuum region of at least 6 Å. The Hartree potential was evaluated on a larger grid with at least 100 Å vacuum around the system and a coarser grid spacing of 1.2 Å, and subsequently refined to the original grid. For the time propagation, we used a time step of Δ*t* = 15 as and total propagation time of at least *T* = 30 fs. The spectra were broadened using Lorentzian spectral broadening with 0.1 eV corresponding to a full width at half-maximum of 0.2 eV. The ground state total energy calculations were calculated using the finite difference mode with wave functions expanded on a real space grid with a mesh spacing of 0.2 Å and a vacuum region around the molecules of 8 Å. The contributions of vdW interactions were evaluated by calculating dispersion corrections as an add-on to standard DFT.^46^ All individual metal nanoparticles and molecules were relaxed independently to the point where all atomic forces are below 0.05 eV/Å, the grid spacing was 0.2 Å. Once relaxed, the dimers and molecule-dimer systems were assembled, but not relaxed further.

### Fitting of Absorption Spectra

All photoabsorption spectra were fitted with the velocity-coupled harmonic oscillator model
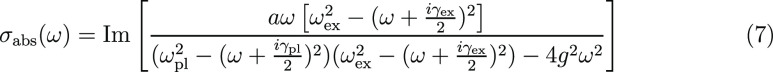
7to obtain the coupling strength *g* as well as the resonance positions and widths of plasmon and molecular excitation, ω_pl_, γ_pl_, ω_ex_, and γ_ex_, respectively. The model assumes that the entire oscillator strength is given by the uncoupled plasmon with amplitude *a*. This is a good approximation, as the molecular photoabsorption spectra are about 10 to 100 times less intense than those of the nanoparticle dimers. This assumption allows us to obtain coupling strengths directly from a single calculation without overfitting.

### Quasinormal Modes

The QNMs of nanosphere dimers are calculated using an approach stemming from the QNMEig solver described in ref (39), which is here based on the Wave Optics Module of COMSOL, a commercial software implementing finite-element method for electromagnetic modeling. The solver allows for the finding of QNM frequencies and normalized QNM fields for dispersive materials with a Drude–Lorentz permittivity. Here, we use a fitted Drude model to match the absorption spectrum of single clusters calculated with TDDFT, effectively constructing an effective, size-dependent permittivity for each metal cluster/particle. The nanoparticle radii *r* correspond to half of the maximum distance between centers of opposite sides of icosahedral (Na, Al) or truncated octahedra (Mg) clusters modeled with TDDFT. The parameters of the Drude permittivity models and nanosphere radii are given in Table 1. In the calculations we use an extra fine mesh setting for the simulation domain and override the settings for spherical domains representing nanoclusters so that the maximum mesh element size is *r*/3, while the minimal mesh element size is *r*/6. We calculate the first 8 eigenfrequencies around the bright dipole mode frequency, which is obtained by modeling nanosphere dimers using SMUTHI, a T-matrix method code.^60^ The normalized fields are then used to calculate the mode volume^8^ for dipole positions in the center of the gap (center of molecule), shifted to 1/4th of the gap and at the position of the end of a molecule. Only the bright dipolar mode contributes, since higher order (e.g., quadrupole) modes are negligibly small for the considered sphere sizes, while the on-axis component of the mode field vanishes at the dimer axis for other dipole modes.

### Software Used

DFT calculations were carried out using the GPAW package^61,62^ with localized basis sets (LCAO mode)^63^ and with uniform real-space grids with the finite difference approximation. TDDFT calculations were conducted using the LCAO-RT-TDDFT implementation in GPAW.^64^ The ASE library^65^ was used for constructing the atomic structures. The NumPy,^66^ SciPy,^67^ and Matplotlib^68^ Python packages, and Inkscape were used for processing data and generating figures.

## Supporting Information Available

Figures S1–S4: Additional modeling details, structures, calculation and fitting results, induced electric fields; Figures S5–S6: Additional energy dependencies on structure parameters; Figures S7–S8: QNM volume calculation results; Tables S1 and S2: Modeled systems and fitted coupling strengths; Supplementary Note S1: Additional discussion of Figure S4 on mode profiles and induced fields; Supplementary Note S2: Derivation of the longitudinal QED Hamiltonian; Supplementary Note S3: Canonical transformation of subsystems (PDF)

## Notes

The atomic structures, the photoabsorption spectra, and basic codes are available at 10.5281/zenodo.3985706. Other data that support the findings of this study are available from the authors upon reasonable request.
