# Supplementary material for: Ultrastrong Coupling of a Single Molecule to a Plasmonic Nanocavity: A First-Principles Study
Source: ACS Photonics. 2022 Mar 2;9(3):1065–77. doi: 10.1021/acsphotonics.2c00066 (PMC8931765; doi:10.1021/acsphotonics.2c00066)
Supplement: Supplementary file 1 — ph2c00066_si_001.pdf [file ph2c00066_si_001.pdf]

Supporting Information

Ultra-strong coupling of a single molecule to a plasmonic nanocavity: A first-principles study

Mikael Kuisma, Benjamin Rousseaux, Krzysztof M. Czajkowski, Tuomas P. Rossi, Timur Shegai, Paul Erhart, and Tomasz J. Antosiewicz

Contents

Figures **2**

S1. DFT structures . . . 2
S2. Photoabsorption spectra . . . 2
S3. Coupling strengths . . . 3
S4. Mode profile and field enhancement profiles . . . 4
S5. Predicted ground state modification . . . 4
S6. Comparison of PBE-DFT, vdW, and USC energy changes for a Mg_{201} dimer coupled to tetracene . . . 5
S7. Theoretical and effective mode volume . . . 6
S8. Quasi-normal mode volumes . . . 6

Tables **7**

S1. Summary of modelled systems . . . 7
S2. Summary of calculated coupling strengths . . . 7

Supplementary Notes **7**

S1. Mode profiles and induced fields of Mg_{201} dimers . . . 7
S2. Longitudinal QED Hamiltonian . . . 8
S3. Canonical Transformation of Subsystems . . . 11

Figures

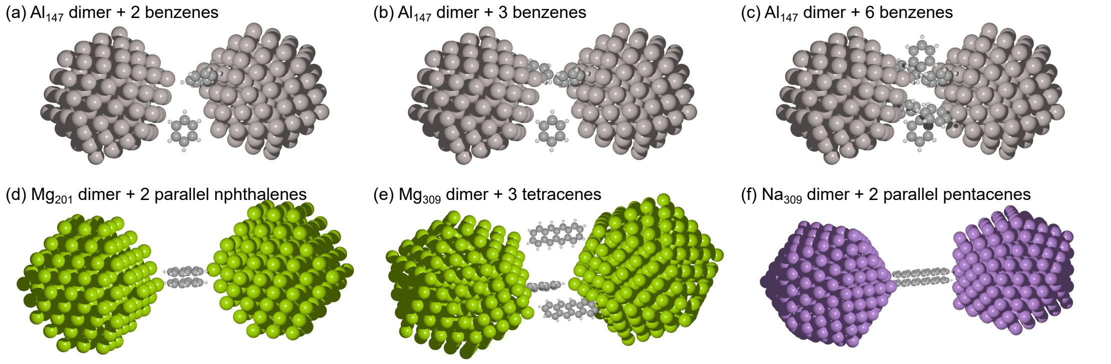

Figure S1: DFT structures. Exemplary systems of metal nanodimers coupled with molecules. (a-c) Dimer of Al_{147} icosahedral particles with 2–6 benzene molecules in the gap. The molecules are arranged in a circular pattern in the middle of the gap between the dimer elements. The radius of this pattern is that of a circle circumscribing any one of the facets of the icosahedral particle. For the Al_{309} dimer (not shown here) the arrangement is the same except for the bigger facets of the larger Al cluster. (d) Dimer of two Mg_{201} regular truncated octahedral particles with two parallel naphthalene molecules placed in the geometrical center of the gap. (e) Dimer of two Mg_{309} icosahedral particles with 3 benzene molecules. This system was modelled with up to 6 molecules in the gap which were arranged the same way as for Al_{147}. (f) Dimer of two Na_{309} icosahedral particles with two parallel pentacene molecules in the geometrical gap.

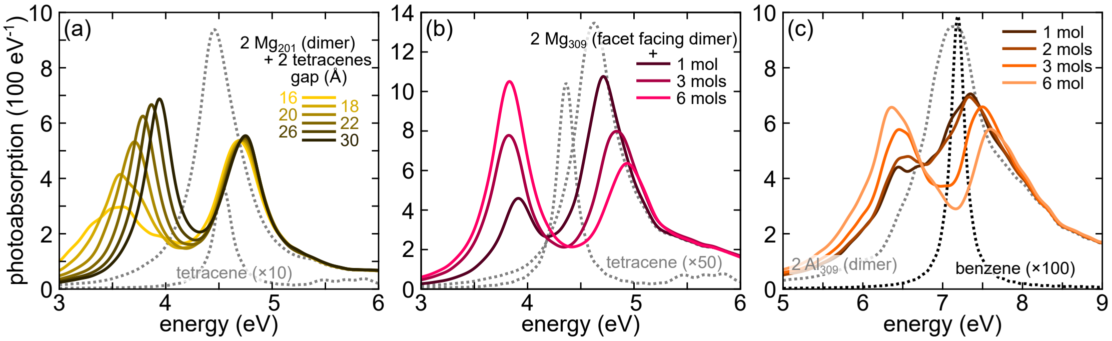

Figure S2: Photoabsorption spectra. Additional photoabsorption spectra of metal dimers strongly coupled to tuned molecules (solid lines), gray dotted lines mark reference spectra of the metal dimers and molecules alone: (a) Mg_{201} nanodimers coupled to two tetracene molecules, (b) Mg_{309} dimers coupled to 1, 3, and 6 tetracene molecules, (c) Al_{309} dimers with 1, 2, 3, and 6 benzene molecules.

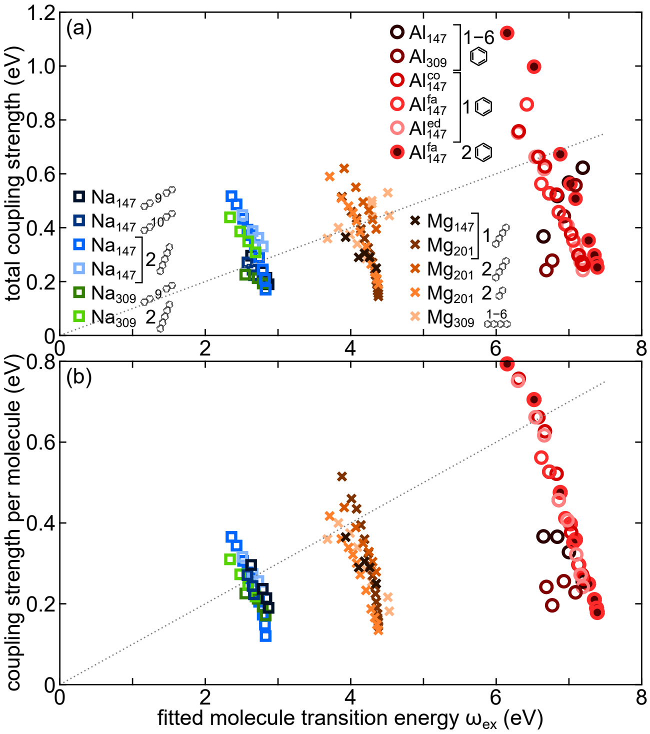

Figure S3: Coupling strengths. Fitted (a) total and (b) per-molecule coupling strength g for all modelled systems plotted versus the fitted molecular transition energy ω_{ex}. The results are arranged in three groups with squares marking Na dimers, crosses Mg dimers, and circles Al dimers. The legend's cartoon insets mark the different modelled cases and for a single shape/color the varying parameter is either the gap size (continuous variation of points) or number of molecules (scattered points), as listed in Table 1. The dotted line marks 10% of the transition energy, which is the assumed limit defining the ultra-strong coupling regime. Results show a significant variation of the coupling strength with the gap size. In all cases the systems can enter the ultra-strong coupling regime.

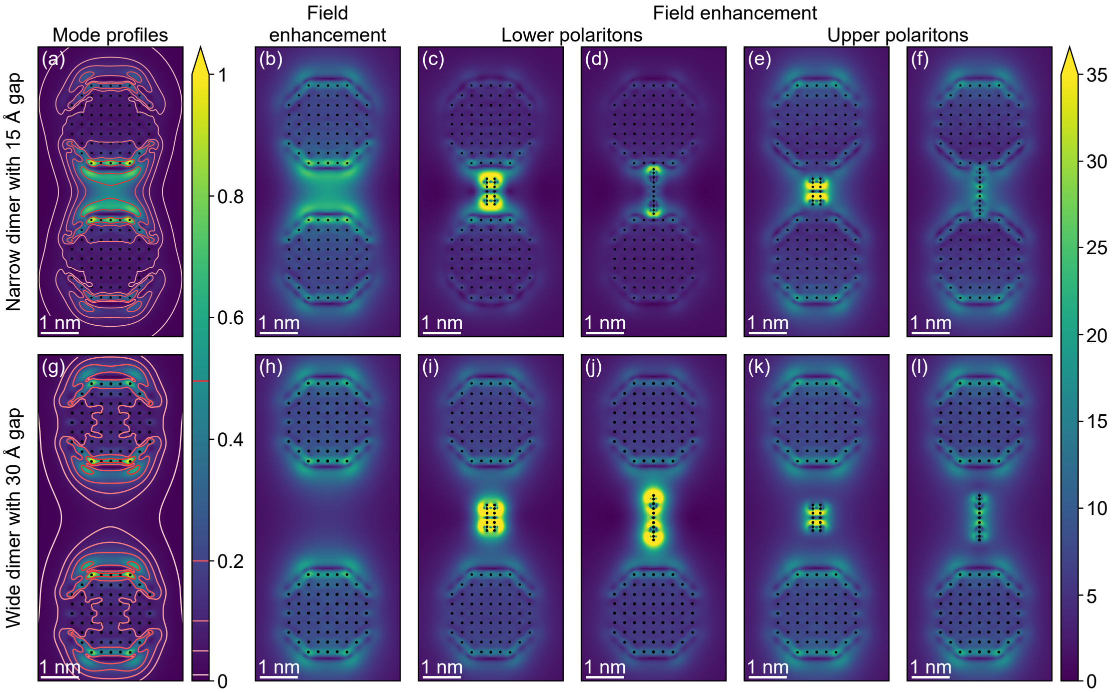

Figure S4: Mode profile and field enhancement profiles. Bright dipolar mode profiles (energy density of the induced electric field) and field enhancements for a Mg_{201} dimer with (a-f) a 15 Å gap and (g-l) a larger 30 Å gap. (a,g) Mode profiles and (b,h) field enhancement for dimers with 15 Å and 30 Å gaps at the gap plasmon energy, respectively. (c-f,i-l) Field enhancements for upper and lower polaritons show that the molecule significantly amplifies the electric field around it and modifies the mode profile and its volume, effectively increasing the coupling strength beyond the efficiency dictated by the mode profile of an empty cavity.

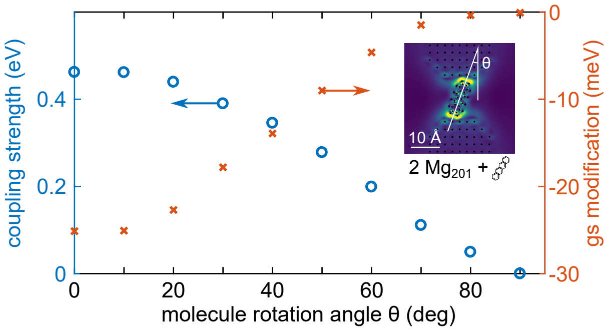

Figure S5: Predicted ground state modification. Coupling strength (circles) and resulting ground state modification (crosses) for Mg_{201} dimers and a single tetracene molecule. The tetracene molecule is rotated while remaining in the center of the gap and the coupling strength decreases from ca. 0.5 eV to 0. The predicted ground state modification correspondingly decreases from -25 meV to 0.

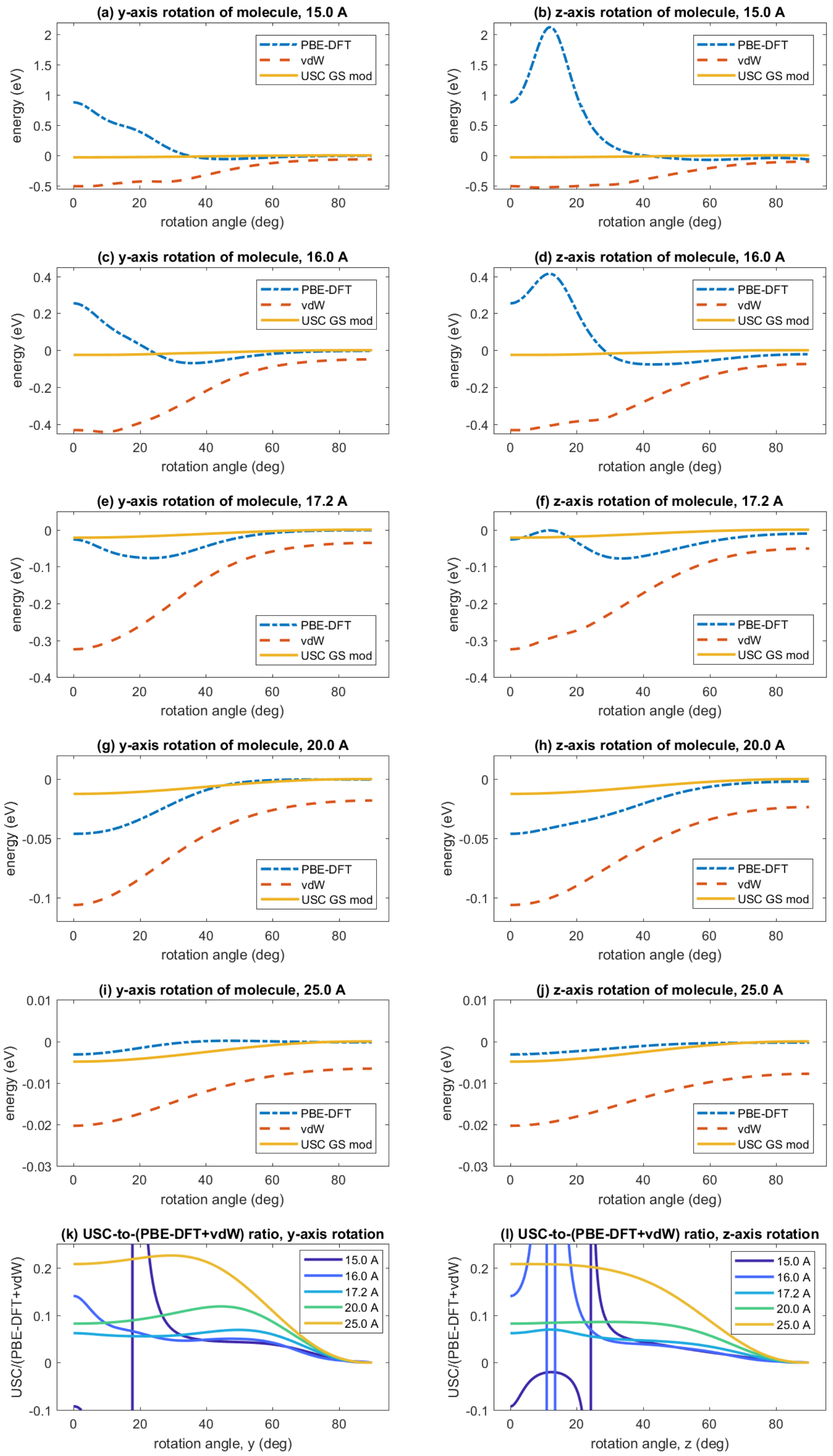

Figure S6: Comparison of PBE-DFT, vdW, and USC energy changes for a Mg_{201} dimer coupled to tetracene . (a-j) PBE-DFT, vdW, and USC energies for y- and z-axis rotation (left and right columns, respectively). (k-l) USC-to-(PBE-DFT+vdW) energy change ratio for different gap sizes.

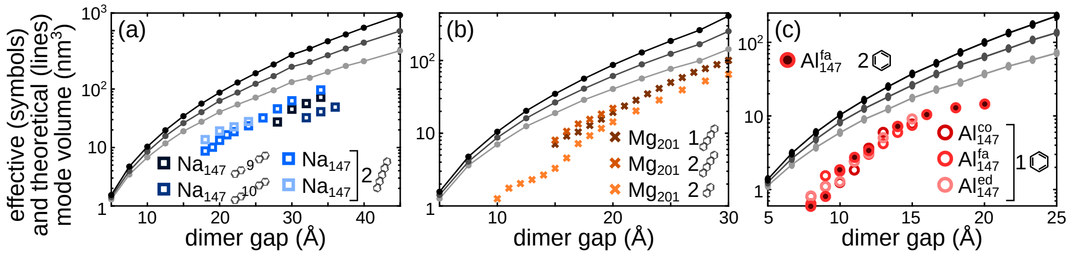

Figure S7: Theoretical and effective mode volume. (a) Sodium dimers with nonacene/decacene and pentacene dimers. (b) Magnesium dimers with tetracene and naphthalene. (c) Aluminum dimers with benzene. The solid lines mark the QNM volumes calculated for dipoles placed in the center (black), $1/3^{rd}$ (gray), and $1/4^{th}$ of the gap (light gray). The symbols in each plot mark the effective mode volumes of each modelled case calculated according to Eq. (3).

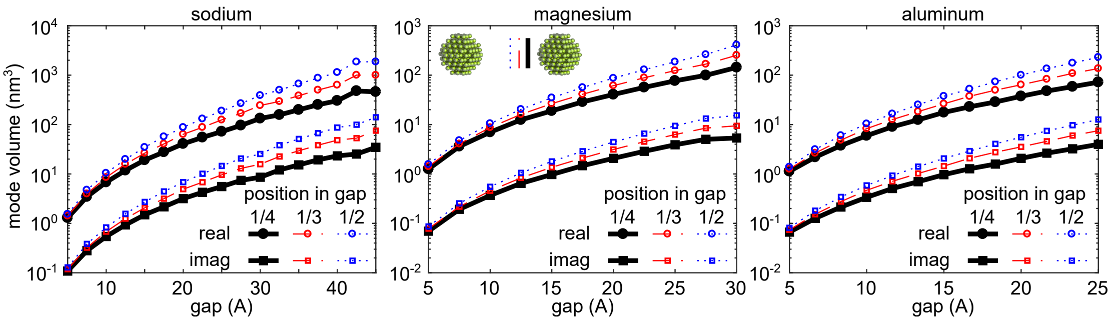

Figure S8: Quasi-normal mode volumes. Comparison of the real and imaginary parts of the position-dependent mode volumes calculated for the test dipole coupling to the dominant dipolar bonding mode. The dipole position is in the middle of the gap (position = 1/2) and displaced toward one the the dimer elements in 1/3 and 1/4 of the gap. In all cases the imaginary part of the mode volume is much smaller, at least one order of magnitude, than the real part. The inset in the middle column marks schematically the positions of the test dipole in the gaps.

Tables

Table S1: Summary of modelled systems. List of modelled systems sorted by atom, structure, gap size, molecule and number N. The molecules are placed in the center of the gap between the dimer components. For Mg and Na for $N = 2$ the molecules are placed parallel to each other in the center of the dimer gap and are separated by 2 Å to ensure a slightly red detuned transition with respect to the plasmon. For Al for $N > 2$ the molecules are placed between the corners of the parallel facets and for $N = 6$ also between the edges.

Atom	Structure	Dimer gap (Å)	Molecule (N)	μ_1 (D)
Al	Al_{147}	8–15	benzene (1)	4.3
		8–20	benzene (2)	
	Al_{147}, Al_{309}	10	benzene (1–6)	
Mg	Mg_{147}	15–20	tetracene (1)	11.1
	Mg_{201}	15–30	tetracene (1)	11.1
		15–20	tetracene (2)	11.1
		10–20	naphthalene (2)	3.9
	Mg_{309}	15–20	tetracene (1–6)	11.1
Na	Na_{147}	28–34	nonacene (1)	15.2
		32–36	decacene (1)	15.3
		18–34	pentacene (2)	11.8
	Na_{309}	28–34	nonacene (1)	15.2
		18–24	pentacene (2)	11.8

Table S2: Summary of calculated coupling strengths. Maximum coupling efficiencies ζ, in the coherent (ζ_N, for $N > 1$), the per-molecule $\zeta_N/\sqrt{N}$, and single-molecule ζ_1 cases.

Atom	Structure	Molecule	ζ_N	$\zeta_N/\sqrt{N}$	ζ_1
Al	Al_{147}	1–6 benzenes	0.182	0.129	0.133
	Al_{309}	1–6 benzenes	0.078	0.037	0.036
Mg	Mg_{147}	1 tetracene	–	–	0.093
	Mg_{201}	1–2 tetracenes	0.159	0.112	0.133
		2 naphthalenes	0.159	0.112	–
	Mg_{309}	1–2 tetracenes	0.117	0.064	0.104
Na	Na_{147}	1 nonacene	–	–	0.113
		1 decacene	–	–	0.105
		2 pentacenes	0.219	0.155	–
	Na_{309}	1 nonacene	–	–	0.088
		2 pentacenes	0.178	0.126	–

Supplementary Notes

Supplementary Note S1: Mode profiles and induced fields of Mg_{201} dimers. The amplifying effect of the molecule on the coupling strength efficiency is supported by the induced fields presented in Fig. S4. Specifically, the fact that the molecule couples to the cavity mode with greater efficiency than predicted by the vacuum field of the bare cavity is confirmed by an analysis of the mode profiles and cross sections of the enhanced induced electric field plotted in Fig. S4 for Mg_{201} dimers with 15 and 30 Å gaps. In the former case in Fig. S4a, the mode is confined to the gap, but its amplitude is on the order of 0.3-0.4 and only very close to the dimers does it exceed 0.5. However, for two naphthalene molecules we obtain effective vacuum fields

which are ca. 3 times larger than the theoretical one and ca. 2 times larger for for tetracene (ratio of the effective and theoretical mode volumes plotted in Fig. 4). In all cases the effective vacuum fields are higher than the ones based on the QNMs.

These increases of the effective vacuum fields are the result of a modification of the cavity's mode volume due to the molecule [1]. This change can be understood in terms of a logical split of the molecule into the electronic HOMO-LUMO transition, which couples to the gap plasmon, and the higher-energy transitions which modify the cavity mode. The result of this can be illustrated by plotting the induced electric field enhancements in Fig. S4 for the bare dimers at the cavity resonance and at the upper and lower polaritons. The addition of molecules to the gap modifies the electric field distribution by focusing its intensity around the molecule. This amplifies the energy density in the gap and results in enhanced coupling beyond what is predicted based on the mode profile of the empty cavity [1]. For the small gap of 15 Å the field enhancement is larger for naphthalene in the gap than for tetracene, what is consistent with the larger amplification of the vacuum vield for the former case. In the 30 Å-gap dimer the field enhancement for both molecules is similar, what is consistent with the equal effective vacuum fields. These results highlight the importance of considering the molecules themselves as equivalent participants to nano- and picoscale cavities in coupling phenomena. Indeed, we observe that molecules may alter small cavities to such large degree, that their size may begin to contribute a large or dominant part of a cavity's mode volume, Fig. S7.

Supplementary Note S2: Longitudinal QED Hamiltonian.

Electrostatic interaction. We consider the system of a single molecule placed at the center of a dimer nanocluster gap (see Fig. S9). The general Hamiltonian description of this system is the minimal coupling Hamiltonian [2]:

$$H = \sum_i \frac{\left(\mathbf{p}_i - e\mathbf{A}_\perp(\mathbf{r}_i)\right)^2}{2m_i} + V_C + \frac{\epsilon_0\epsilon}{2}\int \mathrm{d}^3r \left(\mathbf{E}_\perp^2(\mathbf{r}) + c^2\mathbf{B}^2(\mathbf{r})\right), \tag{S1}$$

where $\mathbf{p}_i, m_i, \mathbf{r}_i$ are the momenta, masses and positions of single charges and V_C is the Coulomb potential taking longitudinal, short-range electromagnetic interaction between charges into account. The effect of transverse electromagnetic radiation is here accounted by the potential vector $\mathbf{A}_\perp(\mathbf{r}_i)$, which induces a coupling term and a self-energy term with the transverse field. However, as risen in refs. [3, 4], the effects of transverse fields should arise when the size of the system is comparable to $ƛ = \hbar c/E$, E being the resonant energy. For $1 < E < 10$ eV, we have $124 < ƛ < 1240$ nm, with in our case is always much larger than the size of the dimer. We can then neglect the effects of the transverse radiation and the Hamiltonian reduced to:

$$H \approx \sum_i \frac{\mathbf{p}_i^2}{2m_i} + V_C. \tag{S2}$$

When considering our two-moieties system (molecule and NP dimer) with only induced dipole moments, one can decompose the Hamiltonian into:

$$H = H_{c1}^0 + H_{c2}^0 + H_m^0 + V_{\mathrm{int}}, \tag{S3a}$$

$$H_{ck}^0 = \sum_{i\in ck} \frac{\mathbf{p}_i^2}{2m_i} + \sum_{i,j\in ck} V_{ij}, \tag{S3b}$$

$$H_m^0 = \sum_{i\in m} \frac{\mathbf{p}_i^2}{2m_i} + \sum_{i,j\in m} V_{ij}, \tag{S3c}$$

$$V_{\mathrm{int}} = \sum_{k=1,2}\sum_{i\in ck}\sum_{j\in m} V_{ij}, \tag{S3d}$$

where ck labels the kth nanocluster, m labels the molecule, and the Coulomb interaction V_C is split into "self" terms summed over charges within each moiety and an interacting term V_{int} including interactions between charges of different moieties.

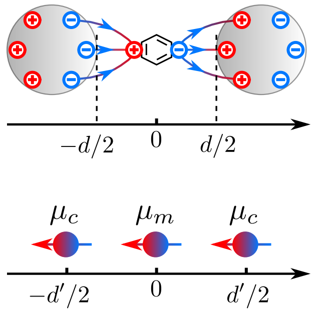

Figure S9: System of a single molecule (as an example: benzene) located in the gap center of a nanoparticle dimer. The gap of length d is defined as the minimal surface-to-surface distance in between the nanoparticles. Below: effective 3-point-dipole modeling of the system. The molecule's point-dipole with moment μ_m is placed between two identical point-dipoles associated with each nanoparticle, with moments μ_c. The effective distance d' is larger than d but smaller than $d+2R$, R being the radius of a single nanoparticle.

If we now assume a dipolar polarization for the nanoclusters and the molecule, we can map the problem to a three-coupled-dipole system where each cluster has an effective dipole moment μ_c (see fig. S9). Modeling the charge oscillations as harmonic oscillators, the Hamiltonian becomes:

$$H = H^0_{c1} + H^0_{c2} + H^0_m + V_{\text{int}}, \tag{S4a}$$
$$H^0_{ck} \approx \omega_c \left(p_k^\dagger p_k + \tfrac{1}{2}\right), \tag{S4b}$$
$$H^0_m \approx \omega_m \left(b^\dagger b + \tfrac{1}{2}\right), \tag{S4c}$$
$$V_{\text{int}} = V_{c1,m} + V_{c2,m} + V_{c1,c2}, \tag{S4d}$$

where here ω_c is the resonance frequency of a single nanocluster with operators obeying $[p_k, p^\dagger_{k'}] = \delta_{kk'}$, ω_m the transition frequency of the molecule with operators obeying $[b, b^\dagger] = 1$, and we have decomposed the induced dipole Coulomb interaction terms $V_{\alpha\beta}$, $\alpha, \beta = c1, c2, m$:

$$V_{\alpha\beta} = \frac{1}{4\pi\epsilon_0\epsilon} \frac{R^2_{\alpha\beta}\boldsymbol{\mu}_\alpha \cdot \boldsymbol{\mu}_\beta - 3(\boldsymbol{\mu}_\alpha \cdot \mathbf{R}_{\alpha\beta})(\boldsymbol{\mu}_\beta \cdot \mathbf{R}_{\alpha\beta})}{R^5_{\alpha\beta}} \tag{S5}$$

where $\mathbf{R}_{\alpha\beta} = \mathbf{r}_\alpha - \mathbf{r}_\beta$, $\mathbf{r}_\alpha$ being the position of the dipole α. Considering now the simple situation where all dipoles are aligned, the molecule is placed at the origin and each cluster dipole is placed at a distance $d'/2$ of the molecule, the Hamiltonian reads:

$$\begin{aligned} H = \omega_c \left(p_1^\dagger p_1 + \tfrac{1}{2}\right) + \omega_c \left(p_2^\dagger p_2 + \tfrac{1}{2}\right) + \omega_m \left(b^\dagger b + \tfrac{1}{2}\right) \\ - \frac{\mu_c^2}{2\pi\epsilon_0\epsilon d'^3}\left(p_1^\dagger + p_1\right)\left(p_2^\dagger + p_2\right) + \frac{\mu_c\mu_m}{2\pi\epsilon_0\epsilon(d'/2)^3}\left(b^\dagger + b\right)\left(p_1^\dagger + p_1 - p_2^\dagger - p_2\right). \end{aligned} \tag{S6}$$

This expression enables to isolate the effective dimer Hamiltonian and diagonalize it to derive the effective coupling strength between the dimer and the molecule.

Diagonalization of the dimer part. Considering now only the dimer part:

$$H_d = \omega_c \left(p_1^\dagger p_1 + \tfrac{1}{2}\right) + \omega_c \left(p_2^\dagger p_2 + \tfrac{1}{2}\right) - \frac{\mu_c^2}{2\pi\epsilon_0\epsilon d'^3}\left(p_1^\dagger + p_1\right)\left(p_2^\dagger + p_2\right), \tag{S7}$$

we can define the Hopfield operators [5]:

$$\Pi_\pm = x_\pm p_1 + y_\pm p_1^\dagger + m_\pm p_2 + h_\pm p_2^\dagger, \tag{S8}$$

with the Hopfield coefficients satisfying the condition $|x_\pm|^2 - |y_\pm|^2 + |m_\pm|^2 - |h_\pm|^2 = 1$ to ensure the bosonicity of the new operators. The latter enters the eigenvalue problem $[\Pi_\pm, H_d] = \omega_\pm \Pi_\pm$

that can be stated in a matrix form:

$$\begin{pmatrix} \omega_c & 0 & g_c/2 & -g_c/2 \\ 0 & -\omega_c & g_c/2 & -g_c/2 \\ g_c/2 & -g_c/2 & \omega_c & 0 \\ g_c/2 & -g_c/2 & 0 & -\omega_c \end{pmatrix} \begin{pmatrix} x_\pm \\ y_\pm \\ m_\pm \\ h_\pm \end{pmatrix} = \omega_\pm \begin{pmatrix} x_\pm \\ y_\pm \\ m_\pm \\ h_\pm \end{pmatrix}, \tag{S9}$$

where we set $g_c = -\mu_c^2/(\pi\epsilon_0\epsilon d'^3)$. Diagonalizing the Hopfield matrix analytically yields the Hopfield coefficients as well as the eigenfrequencies:

$$\omega_\pm = \omega_c\sqrt{1 \pm g_c/\omega_c}. \tag{S10}$$

These two eigenfrequencies corresponds to two eigenmodes: the lower energy mode ω_- is the one described in fig. S9, where the two dipoles are aligned and oscillate in phase ($|+-+-\rangle$) while the higher energy mode is a "dark" contribution where the dipoles oscillates with a phase of π ($|-++-\rangle$). Therefore, we identify the dimer eigenfrequency as $\omega_d \equiv \omega_-$, which is the peak seen in e.g. photoabsorption spectra. It is then expected that $\omega_d < \omega_c$, which is clearly seen by looking at e.g. an Al_{309} cluster ($\omega_c \approx 7.7$ eV [6]) and a $2Al_{309}$ dimer ($\omega_d \approx 7.2$ eV). Using (S10), we can even deduce the coupling strength g_c between the clusters as:

$$g_c = \frac{\omega_c^2 - \omega_d^2}{\omega_c}. \tag{S11}$$

In addition, if the single cluster dipole moment μ_c is known, one can then find the effective distance d' between the two point dipoles associated with each cluster.

The remaining part of the diagonalization procedure consists in inverting the transformation:

$$\begin{pmatrix} \Pi_+ + \Pi_+^\dagger \\ \Pi_- + \Pi_-^\dagger \end{pmatrix} = \begin{pmatrix} a_+ & b_+ \\ a_- & b_- \end{pmatrix} \begin{pmatrix} p_1 + p_1^\dagger \\ p_2 + p_2^\dagger \end{pmatrix}, \tag{S12}$$

where $a_\pm = x_\pm + y_\pm$ and $b_\pm = m_\pm + h_\pm$. Solving for the Hopfield coefficients, we find $a_\pm = \sqrt{\omega_\pm/(2\omega_c)}$ and $b_\pm = \pm\sqrt{\omega_\pm/(2\omega_c)}$. This allows for writing the operator $p_1^\dagger + p_1 - p_2^\dagger - p_2$ in terms of the new operators in (S6) and we find:

$$\begin{aligned} H = {} & \omega_- \left(\Pi_-^\dagger \Pi_- + \tfrac{1}{2}\right) + \omega_+ \left(\Pi_+^\dagger \Pi_+ + \tfrac{1}{2}\right) + \omega_m \left(b^\dagger b + \tfrac{1}{2}\right) \\ & - \frac{\mu_c \mu_m}{2\pi\epsilon_0\epsilon(d'/2)^3}\sqrt{\frac{2\omega_c}{\omega_-}}\left(b^\dagger + b\right)\left(\Pi_-^\dagger + \Pi_-\right) + \frac{\mu_c \mu_m}{2\pi\epsilon_0\epsilon(d'/2)^3}\sqrt{\frac{2\omega_c}{\omega_+}}\left(b^\dagger + b\right)\left(\Pi_+^\dagger + \Pi_+\right). \end{aligned} \tag{S13}$$

Eigenfrequencies of the dimer-molecule system. Considering the case where the molecule is nearly resonant with the bright dimer mode $\omega_d \equiv \omega_-$, the dark mode can be neglected and we have:

$$H = \omega_d \left(B^\dagger B + \tfrac{1}{2}\right) + \omega_m \left(b^\dagger b + \tfrac{1}{2}\right) + g_{md}\left(b^\dagger + b\right)\left(B^\dagger + B\right), \tag{S14}$$

where $B \equiv \Pi_-$ and $g_{md} = -\frac{\mu_c\mu_m}{2\pi\epsilon_0\epsilon(d'/2)^3}\sqrt{\frac{2\omega_c}{\omega_d}}$ is the coupling strength between the bright mode and the molecule exciton. This Hamiltonian can again be diagonalized by introducing new polaritonic operators:

$$P_\pm = \xi_\pm b + \upsilon_\pm b^\dagger + \mu_\pm B + \nu_\pm B^\dagger, \tag{S15}$$

with the new Hopfield coefficients satisfying $|\xi_\pm|^2 - |\upsilon_\pm|^2 + |\mu_\pm|^2 - |\nu_\pm|^2 = 1$. The diagonal form is:

$$H = \Omega_+ \left(P_+^\dagger P_+ + \tfrac{1}{2}\right) + \Omega_- \left(P_-^\dagger P_- + \tfrac{1}{2}\right), \tag{S16}$$

with the analytical eigenfrequencies being given by the biquadratic equation:

$$\left(\Omega_\pm^2 - \omega_d^2\right)\left(\Omega_\pm^2 - \omega_m^2\right) - 4g_{md}^2\omega_d\omega_m = 0, \tag{S17}$$

yielding:

$$\Omega_\pm = \frac{1}{\sqrt{2}}\sqrt{\omega_d^2 + \omega_m^2 \pm \sqrt{(\omega_d^2 - \omega_m^2)^2 + 16g_{md}^2\omega_d\omega_m}}. \tag{S18}$$

The ground state modification is then given by:

$$\Delta E_{GS} = \frac{\hbar}{2}\left(\Omega_+ + \Omega_- - \omega_d - \omega_m\right). \tag{S19}$$

When the system is on resonance $\omega_m = \omega_d \equiv \omega$, then:

$$\Omega_\pm = \omega\sqrt{1 \pm 2g_{md}/\omega} = \omega\left(1 \pm \frac{g_{md}}{\omega} - \frac{g_{md}^2}{2\omega^2} + \mathcal{O}\left(\frac{g_{md}^3}{\omega^3}\right)\right). \tag{S20}$$

The first correction to the ground state energy shift is then:

$$\Delta E_{GS} \approx -\frac{g_{md}^2}{2\omega} = -\frac{\mu_c^2\mu_m^2\omega_c}{4\hbar\pi^2\epsilon_0^2\omega^2\epsilon^2(d'/2)^6}, \tag{S21}$$

which then corresponds to the London force (van der Waals between induced dipoles) in $1/R^6$ [4]. The van der Waals interaction can then be interpreted from the point of view of USC regime physics.

Supplementary Note S3: Canonical Transformation of Subsystems.

The direct random phase approximation Hamiltonian (see e.g. ref. [7]) coupling plasmonic (P) and molecular (M) subsystems is

$$\hat{H}_1^{\mathrm{RPA}} = E_0 + \frac{1}{2}\begin{bmatrix}\vec{a}_P^\dagger\\ \vec{a}_M^\dagger\\ \vec{a}_P\\ \vec{a}_M\end{bmatrix}^T \begin{bmatrix}\Delta_{PP}+K_{PP} & K_{PM} & K_{PP} & K_{PM}\\ K_{MP} & \Delta_{MM}+K_{MM} & K_{MP} & K_{MM}\\ K_{PP} & K_{PM} & \Delta_{PP}+K_{PP} & K_{PM}\\ K_{MP} & K_{MM} & K_{MP} & \Delta_{MM}+K_{MM}\end{bmatrix}\begin{bmatrix}\vec{a}_P\\ \vec{a}_M\\ \vec{a}_P^\dagger\\ \vec{a}_M^\dagger\end{bmatrix} - E_{\mathrm{self}}, \tag{S22}$$

where $\Delta_{ia,i'a'} = \delta_{ii'}\delta_{aa'}(\epsilon_a - \epsilon_i)$ contains non-interacting Kohn–Sham excitation energies in its diagonal and

$$K_{ia,i'a'} = \int \mathrm{d}\boldsymbol{r}\int \mathrm{d}\boldsymbol{r}'\,\frac{\psi_i(\boldsymbol{r})\psi_a(\boldsymbol{r})\psi_{i'}(\boldsymbol{r}')\psi_{a'}(\boldsymbol{r}')}{4\pi\epsilon_0|\boldsymbol{r}-\boldsymbol{r}'|} \tag{S23}$$

contains the Coulomb matrix elements. We can first transform into generalized position and momentum representation,

$$\hat{H}_2^{\mathrm{RPA}} = \frac{1}{2}\begin{bmatrix}\vec{Q}_P\\ \vec{Q}_M\\ \vec{P}_P\\ \vec{P}_M\end{bmatrix}^T \begin{bmatrix}\Delta_{PP}+2K_{PP} & 2K_{PM} & 0 & 0\\ 2K_{MP} & \Delta_{MM}+2K_{MM} & 0 & 0\\ 0 & 0 & \Delta_{PP} & 0\\ 0 & 0 & 0 & \Delta_{MM}\end{bmatrix}\begin{bmatrix}\vec{Q}_P\\ \vec{Q}_M\\ \vec{P}_P\\ \vec{P}_M\end{bmatrix}, \tag{S24}$$

We then diagonalize the Casida equations for the individual subsystems as $O_P^T\Omega_P^2 O_P = \Delta_P^2 + 2\sqrt{\Delta_P}K_{PP}\sqrt{\Delta_P}$, for M respectively and perform a following canonical transformation

$$\vec{Q}_P = \Delta_P^{1/2}O_P\Omega_P^{-1/2}\vec{Q}'_P \tag{S25}$$

$$\vec{Q}_M = \Delta_M^{1/2}O_M\Omega_M^{-1/2}\vec{Q}'_M \tag{S26}$$

$$\vec{P}_P = \Delta_P^{-1/2}O_P\Omega_P^{1/2}\vec{P}'_P \tag{S27}$$

$$\vec{P}_M = \Delta_M^{-1/2}O_M\Omega_M^{1/2}\vec{P}'_M, \tag{S28}$$

which results into a following Hamiltonian matrix

$$\begin{bmatrix}\Omega_P & 2\Omega_P^{-1/2}O_P^T\Delta_P^{1/2}K_{PM}\Delta_M^{1/2}O_M\Omega_M^{-1/2} & 0 & 0\\ 2\Omega_M^{-1/2}O_M^{-1}\Delta_M^{1/2}K_{MP}\Delta_P^{1/2}O_P^T\Omega_P^{-1/2} & \Omega_M & 0 & 0\\ 0 & 0 & \Omega_P & 0\\ 0 & 0 & 0 & \Omega_M\end{bmatrix} \tag{S29}$$

Let us examine the term $2K'_{PM} = 2\Omega_P^{-1/2}O_P^T\Delta_P^{1/2}K_{PM}\Delta_M^{1/2}O_M\Omega_M^{-1/2}$ closer by adding indices. Let I_P and I_M enumerate Casida eigenvectors for plasmon and molecular system respectively,

and k_P and k_M indices stand for the Kohn-Sham state pairs of plasmon and molecular system respectively, and assuming dipolar coupling via the dipole tensor $V_{cc'}(R)$, where R is the distance vector between the subsystems and $c \in \{x, y, z\}$,

$$\begin{aligned}(K'_{PM})_{I_P I_M} =& \Omega_{I_P}^{-1/2} \sum_{k_P, k_M} O_{I_P k_P} \Delta_{k_P}^{1/2} \\ & \underbrace{\sum_{cc'} \mu_{k_P,c} V_{cc'}(R) \mu_{k_M,c'}}_{K_{PM,k_P k_M}} \Delta_{k_M}^{1/2} O_{I_M k_M} \Omega_{I_M}^{-1/2}.\end{aligned} \tag{S30}$$

The transition dipole moment of a Casida transition is

$$\mu_{I_M,c} = \sum_{k_M} \sqrt{\frac{\Delta_{k_M}}{\Omega_{I_M}}} O_{I_M k_M} \mu_{k_M,c},$$

and thus we may write Eq. S30 as

$$(K'_{PM})_{I_P I_M} = \sum_{cc'} \mu_{I_P,c} V_{cc'}(R) \mu_{I_M,c'},$$

and so we have finally established that subsystems may first be diagonalized, and subsequently coupled, while the only approximation performed is the dipole approximation. Our recent work [8] on dipole coupling plasmonic nanoparticles utilizes this by mutually coupling already diagonalized TDDFT systems. In other words, Casida excitation energies and transition dipole moments are sufficient to exactly couple two subsystems (up to the dipole approximation). Renormalization inside the subsystem sub-block for example due to plasmon lifting the excitation energies are fully accounted for by the diagonalized Casida eigenvectors and dipole moments. There are no special intersystem Coulomb effects beyond the information encoded into the Casida eigenvectors.

References

[1] Yang, Z.-J., Antosiewicz, T. J. & Shegai, T. Role of material loss and mode volume of plasmonic nanocavities for strong plasmon-exciton interactions. *Optics Express* **24**, 20373–20381 (2016).

[2] Cohen-Tannoudji, C., Dupont-Roc, J. & Grynberg, G. *Photons and Atoms-Introduction to Quantum Electrodynamics* (1997).

[3] Power, E. & Zienau, S. On the radiative contributions to the van der waals force. *Il Nuovo Cimento (1955-1965)* **6**, 7–17 (1957).

[4] Holstein, B. R. The van der waals interaction. *American Journal of Physics* **69**, 441–449 (2001).

[5] Todorov, Y. Dipolar quantum electrodynamics theory of the three-dimensional electron gas. *Physical Review B* **89**, 075115 (2014).

[6] Rossi, T. P., Shegai, T., Erhart, P. & Antosiewicz, T. J. Strong plasmon-molecule coupling at the nanoscale revealed by first-principles modeling. *Nat. Commun.* **10**, 3336 (2019).

[7] Scuseria, G. E., Henderson, T. M. & Bulik, I. W. Particle-particle and quasiparticle random phase approximations: Connections to coupled cluster theory. *The Journal of Chemical Physics* **139**, 104113 (2013).

[8] Fojt, J., Rossi, T. P., Antosiewicz, T. J., Kuisma, M. & Erhart, P. Dipolar coupling of nanoparticle-molecule assemblies: An efficient approach for studying strong coupling. *The Journal of Chemical Physics* **154**, 094109 (2021).
